# Depression-induced systemic C16-ceramide deficiency promotes OSCC via attenuation of mitochondrial apoptosis

**DOI:** 10.1007/s00018-026-06267-1

**Published:** 2026-06-06

**Authors:** Yuan Pan, Jingjing Guan, Yueqi Wang, Rongchun Yang, Keyu Lai, Xijuan Chen, Juan Xia

**Affiliations:** 1 Hospital of Stomatology, Sun Yat-sen University, Guangzhou, Guangdong 510055 China; 2 Guangdong Provincial Key Laboratory of Stomatology, Guangzhou, Guangdong 510055 China; 3 Guanghua School of Stomatology, Sun Yat-sen University, Guangzhou, Guangdong 510055 China; 4 Key Laboratory of Stem Cells and Tissue Engineering (Ministry of Education), Zhongshan School of Medicine, Sun Yat-sen University, Guangzhou, 510055 China

**Keywords:** Depression, Oral squamous cell carcinoma, Ceramide, Mitochondrial permeability transition pore, Apoptosis

## Abstract

Depression is a recognized risk factor for cancer progression, but the underlying metabolic mediators remain unclear. Here, we identify circulating C16-ceramide depletion as a key mechanism linking depression to oral squamous cell carcinoma (OSCC) progression. Mendelian randomization analysis first established a genetic causal link between depression and increased head and neck cancer risk. In mice, chronic stress, mimicking depression, accelerated orthotopic tongue tumor growth and concurrently reduced circulating levels of C16-ceramide. Exogenous restoration of C16-ceramide reversed this tumor-promoting effect. Mechanistically, C16-ceramide was taken up by OSCC cells via the scavenger receptor SCARB1, accumulated in mitochondria, and induced opening of the mitochondrial permeability transition pore (mPTP), thereby triggering mitochondrial apoptosis. Inhibition of mPTP opening abolished the anti-tumor effects of C16-ceramide. Clinically, a gene signature reflecting this C16-ceramide–mPTP–apoptosis axis was associated with favorable survival in OSCC patients. Our study reveals a novel metabolic pathway whereby depression promotes oral carcinogenesis by depleting a circulating tumor-suppressive lipid, highlighting the therapeutic potential of targeting the C16-ceramide-mPTP-apoptosis pathway.

**Supplementary Information:**

The online version contains supplementary material available at 10.1007/s00018-026-06267-1.

## Introduction

Head and neck squamous cell carcinoma (HNSCC) is the sixth most common cancer worldwide. In Asia, oral squamous cell carcinoma (OSCC) represents a major subtype with a particularly high incidence, where frequent recurrence and metastasis contribute to its poor prognosis [1, 2]. Despite advances in multimodal therapy, the prognosis for advanced OSCC remains dismal, with five-year survival rates stagnating below 40% [3–5]. This clinical impasse underscores an urgent need to elucidate non-traditional, modifiable drivers of OSCC progression.

Beyond established risk factors such as smoking, alcohol and HPV infection, epidemiological evidence increasingly points to psychosocial stress as a significant contributor to cancer pathogenesis [6, 7]. Depression, a frequent comorbidity in cancer patients, is consistently associated with elevated incidence and mortality across several malignancies, including lung [8], breast [9], and prostate [10] cancers. While chronic stress—a core feature of depression—is known to dysregulate the hypothalamic-pituitary-adrenal axis and promote a pro-inflammatory milieu [11–13], the precise molecular mechanisms translating psychological distress into tumor-promoting signals remain poorly defined. This gap represents a central challenge in psycho-oncology.

Mounting evidence from both clinical cohorts and mechanistic studies suggests that chronic stress and depression induce profound systemic metabolic alterations. These range from broad dysregulation of blood lipids and glucose [14] to specific perturbations in liver-derived metabolites [15] and brain-region-specific cellular metabolism [16]. These circulating metabolic shifts are postulated to act as key biological effectors, transmitting the systemic impact of psychological stress to peripheral tissues, including tumors [17–19]. However, the identity of specific circulating metabolites that functionally link depression to cancer progression is largely unknown.

Within the vast metabolome, sphingolipids emerge as compelling candidates. This class of bioactive lipids plays pivotal roles in cellular stress response, senescence and apoptosis [20–22]. Ceramides, central sphingolipid metabolites, are well-established tumor suppressors capable of halting proliferation and initiating programmed cell death [23–25]. Traditionally, C16-ceramide is known to be produced via de novo synthesis during acute cellular stress, causing mitochondrial dysfunction and triggering apoptosis [26, 27]. Crucially, recent clinical lipidomic studies have identified systemic ceramide dysregulation as a hallmark of psychological distress. Plasma levels of specific ceramides, especially C16-ceramide, are significantly altered in patients with major depressive disorder (MDD) and closely correlate with symptom severity [28–30]. However, prolonged psychological stress creates a fundamentally different metabolic state. While acute stress causes a quick local surge in ceramide to defend the cell, chronic stress—a core feature of depression—leads to metabolic exhaustion. Comprehensive metabolic profiling indicates that this chronic systemic exhaustion forces the body into a general hypometabolic adaptation. Ultimately, this leads to a broad depletion of the circulating sphingolipid pool, including C16-ceramide [31]. Despite its dual role as a depression-sensitive systemic metabolite and a potent intracellular tumor suppressor, the bridging role of C16-ceramide in psycho-oncology remains unexplored. This raises a provocative, yet untested, hypothesis: could depression promote tumorigenesis by systemically depleting a specific circulating tumor-suppressive metabolite, such as C16-ceramide, thereby relieving a critical brake on cancer cell survival?

In this study, we integrate human genetics, metabolomics and functional oncology to systematically dissect the depression–OSCC axis. We first establish a genetic causal link between depression and HNSCC risk using Mendelian randomization. We then identify circulating C16-ceramide deficiency as a key metabolic mediator of this relationship. Through a series of in vivo and in vitro experiments, we demonstrate that exogenous C16-ceramide supplementation reverses depression-accelerated tumor growth and directly induces mitochondrial permeability transition pore (mPTP)-dependent apoptosis in OSCC cells. Furthermore, we provide evidence that scavenger receptor class B type 1 (SCARB1) facilitates the cellular uptake and mitochondrial targeting of C16-ceramide in tumor epithelial cells. Finally, we construct a “C16-mPTP-apoptosis” gene signature that holds prognostic value in OSCC patients. Our findings unveil a novel metabolic pathway at the mind–body interface in cancer, positioning circulating C16-ceramide and its downstream mitochondrial apoptotic pathway as potential therapeutic targets for OSCC, particularly in the context of depression.

## Materials and methods

### Mendelian randomization analysis and data sources

Summary statistics for the plasma metabolome analysis were sourced from the GWAS Catalog at the European Bioinformatics Institute (EBI), encompassing 1 091 metabolites and 309 ratios from 8 299 European individuals (accession numbers GCST90199621 to GCST90201020). Genetic instruments for depression were derived from GWAS summary statistics (accession: GCST90468123), comprising 394 626 participants of European descent. Genetic associations with head and neck cancer, including 373 122 individuals of European ancestry, were retrieved from the UK Biobank (ieu-b-4912). We performed two-sample Mendelian randomization (MR) analyses to evaluate (1) the causal effect of depression on head and neck cancer risk and (2) the potential mediating role of plasma metabolites within this causal pathway. The principal method employed was the IVW method. Additional methods, such as MR‒Egger, weighted median (WM), simple mode, and weighted mode, were employed as supplementary approaches to assess causal effects and correct for horizontal pleiotropy. The sensitivity analyses included Cochran’s Q test for heterogeneity and the MR‒Egger intercept test for pleiotropy. All analyses were performed via the TwoSampleMR package in R.

### Mice

All the mice were purchased from Ruiye Model Animal (Guangzhou) Biotechnology. Animal care and experimental procedures were performed under SPF conditions. For the in vivo administration of C16-ceramide, the compound was delivered via tail vein injection at a dose of 5 mg/kg or 10 mg/kg every 48 h for a total of 7 days prior to plasma sample collection. For the subsequent therapeutic intervention in OSCC-bearing mice, C16-ceramide was administered at the optimized dose of 5 mg/kg via tail vein injection every 48 h throughout the tumor progression phase. All procedures involving mice were conducted in accordance with the guidelines outlined in the National Research Council’s Guide for the Care and Use of Laboratory Animals, as well as the Institutional Animal Care and Use Committee (IACUC), and were approved by the Animal Ethics Committee (AEC) of Ruiye Model Animal (Guangzhou) Biotechnology. (Approval Number: RYEth-20241201817). The procedures also followed the guidelines provided by the Association for Assessment and Accreditation of Laboratory Animal Care International (AAALAC). 6-week-old male C57BL/6J mice were housed under specific pathogen-free conditions with a 12 h light/dark cycle and free access to food and water.

### In vivo tumor models

An orthotopic mouse model of OSCC was established by inoculating MTCQ1 cells into the tongues of 8-week-old male C57BL/6J mice, immediately following the 14-day CRS or control paradigm. Briefly, 3 × 10^6^ cells were resuspended in 50 µL of a 1:1 mixture of PBS and Matrigel matrix and carefully injected into the submucosal layer of the anterior tongue under general anesthesia induced by the intraperitoneal administration of pentobarbital (50 mg/kg). The mice were monitored every 48 h for tumor growth and overall health. The tumor volume was calculated via the formula V = (L × W²)/2, where L represents the longest diameter and W represents the shortest diameter perpendicular to L. All the procedures adhered to predefined humane endpoints. On day 21 post-inoculation, the animals were euthanized, and the tumors and major organs were harvested for subsequent histological evaluation.

### Depression models

Chronic Restraint Stress (CRS) model. The mice were randomly allocated to either the control group or the CRS group. CRS subjects were individually restrained in ventilated, transparent acrylic tubes for 6 h daily (10:00 AM to 4:00 PM). Throughout restraint, each tube was positioned inside the animal’s home cage to preserve ambient and olfactory contextual cues. The control animals were transferred to a clean, empty cage for an equivalent 6-hour period without restraint before being returned to their home cages. The restraint procedure was repeated daily for 14 consecutive days.

Chronic Unpredictable Mild Stress (CUMS) model. The mice were randomly allocated to either the control group or the CUMS group. Mice in the CUMS group were subjected to a sequence of random, unpredictable mild stressors daily for 21 consecutive days. These stressors included food/water deprivation for 24 h, overnight continuous illumination, wet bedding for 24 h, 45° cage tilt for 24 h and swimming in cold water (4 °C) for 5 min. To prevent habituation, the same stressor was never applied on consecutive days. Control mice were housed undisturbed under standard specific-pathogen-free conditions.

A series of behavioral tests—the sucrose preference test, open field test, forelimb grip strength test and tail suspension test—were initiated 24 h after the final restraint session, with a 48-hour interval between each assay. All behavioral evaluations were conducted in a dedicated sound-attenuated testing room by an experimenter blinded to the experimental group assignment.

### Behavioral tests

For the sucrose preference test (SPT), the mice were acclimated to two bottles (water and 1% sucrose) for 24 h, followed by testing after 12 h of water deprivation. Sucrose preference was calculated as sucrose consumption/total fluid intake × 100%.

For the open field test (OFT), the mice were placed in a 40 × 40 cm arena for 5 min. Movement trajectories and time in the center zone were recorded and analyzed via CleverSys/PhenoScan software.

For the tail suspension test (TST), the mice were suspended by the tail for 6 min. Immobility time during the last 4 min was scored automatically by CleverSys/PhenoScan software.

For the forelimb grip strength test, the mice were allowed to grasp the pull-bar grid with their forelimbs and were gently pulled backwards by the tail parallel to the grid until they released their grip. The peak force exerted prior to release was recorded. The test was repeated three times for each mouse with a 5-minute rest interval between trials, and the average maximal force was calculated for statistical analysis.

### Plasma collection and broad-targeted lipidomic analysis

At the end of the experimental interventions, blood samples were collected via retro-orbital sinus bleeding into EDTA-coated tubes. The samples were immediately centrifuged at 3,000 × g for 15 min at 4 °C, and the upper plasma layer was carefully collected, aliquoted, and stored at -80 °C until further analysis.

For lipid extraction, 100 µL of plasma was mixed with 300 µL of pre-chilled methanol and 1 mL of methyl tert-butyl ether (MTBE). The mixture was vortexed for 30 s and sonicated for 15 min at 4 °C. Subsequently, 300 µL of LC-MS grade water was added to induce phase separation. After a 10-min incubation at 4 °C, the mixture was centrifuged at 13,000 × g for 30 min at 4 °C. The upper organic phase was collected, dried under a nitrogen stream, and reconstituted in 200 µL of acetonitrile/isopropanol/water (65:30:5, v/v/v).

Lipidomic profiling was performed using a Waters ACQUITY UPLC system coupled with a SCIEX Triple Quad™ 6500 + mass spectrometer. Chromatographic separation was achieved on an ACQUITY UPLC BEH C18 column (1.7 μm, 2.1 mm × 100 mm) at 40 °C. Mobile phase A consisted of water/acetonitrile (2:3) containing 10 mM ammonium acetate, and mobile phase B consisted of isopropanol/acetonitrile (9:1) containing 10 mM ammonium acetate. Mass spectrometry data were acquired in Multiple Reaction Monitoring (MRM) mode using SCIEX OS software for peak alignment, retention time correction, and peak area extraction. Lipid identification and absolute quantification were performed based on a self-built lipid database (VGDB) and stable isotope internal standards.

### RNA sequencing and bioinformatic analysis

#### Bulk RNA sequencing and analysis

Total RNA was extracted via MagZol Reagent. The PCR products were subsequently purified (AMPure XP system), and library quality was assessed on an Agilent Bioanalyzer 2100 system. Clustering of the index-coded samples was performed on a cBot Cluster Generation System via the TruSeq PE Cluster Kit v3-cBot-HS (Illumina) according to the manufacturer’s instructions. After cluster generation, the library preparations were sequenced on an Illumina NovaSeq X Plus platform, and 150 bp paired-end reads were generated. The DESeq R package (1.18.0) was used for differential expression analysis. The resulting P values were adjusted via Benjamini and Hochberg’s approach for controlling the false discovery rate. Genes with a fold change > 1.5 and a P value < 0.05 were considered differentially expressed. KEGG and GSEA analyses were conducted via clusterProfiler.

#### Single-cell RNA sequencing data analysis

Public scRNA-seq data from OSCC and matched normal tissues were obtained from the Gene Expression Omnibus (GEO) under accession number GSE181919. The dataset included 3 samples of non‑tumoral surrounding normal oral tissue and 10 samples of primary OSCC. Data preprocessing and analysis were performed using the Seurat R package (v5.0.1). Low quality cells expressing fewer than 200 genes or more than 8 000 genes, or with > 10% mitochondrial gene content were filtered out. Genes detected in less than 0.1% of all cells were removed. The SCTransform method was used for normalization and variance stabilization. Batch effects across samples were corrected using Harmony. Dimensionality reduction was performed via principal component analysis (PCA), followed by uniform manifold approximation and projection (UMAP) for two‑dimensional visualization. Cell clusters were identified using Seurat’s FindNeighbors and FindClusters functions and were manually annotated into major lineages (epithelial, immune, endothelial, and mesenchymal cells) based on the expression of canonical marker genes. For pathway analysis, epithelial cells were subset and further classified into SCARB1‑high and SCARB1‑low groups based on their expression level. GSEA was then performed to compare these two groups using the fgsea R package (v1.24.0) and custom-defined gene sets. Enrichment results with a normalized enrichment score (NES) absolute value > 1.0 and a false discovery rate (FDR) q-value < 0.25 were considered significant.

### Cell culture

The human OSCC cell lines (HSC6, CAL27 and CAL33) and the mouse OSCC cell line (MTCQ1) were cultured in Dulbecco’s modified Eagle’s medium (DMEM; Thermo Fisher Scientific, Cat# C11995500BT) supplemented with 10% (v/v) fetal bovine serum (FBS; Vazyme, Cat# F101-01) and 2% penicillin‒streptomycin (NCM Biotech, Cat# C100C5). The cells were maintained at 37 °C in a humidified atmosphere containing 5% CO₂. The HSC6 cell line was specifically used for deeper in vitro mechanistic validations, including the stable target gene knockdown (SCARB1) and the CsA rescue experiments. For drug treatments, the cells were exposed to C16-ceramide at final concentrations of 5, 20, and 50 µM for 24 h. To investigate the role of the mPTP, CsA was administered 30 min prior to C16-ceramide treatment at a concentration of 5 µM.

### Lentiviral construction and stable cell transduction

The short hairpin RNA (shRNA) sequence targeting human *SCARB1* (5’-CCCTTTCTACTTGTCTGTCTA-3’) and the specific shRNA sequence targeting mouse *Scarb1* (5‘-GCTTCCCATAAAGGGCAAAT-3’) were synthesized and inserted into the pLKO.1-Puro vector (Miaoling Plasmid, Cat# P0258) using AgeI and EcoRI restriction sites. Plasmids were propagated in STBL3 competent E. coli cells (AlpalifeBio, Cat# KTSM110L), and endotoxin-free plasmid DNA was prepared using the FastPure EndoFree Plasmid Mini Plus Kit (Vazyme, Cat# DC204-01). Lentiviral particles were produced in HEK293T cells by co-precipitating the target plasmid (shSCARB1 or empty vector shNC) with packaging vectors (psPAX2 and pMD2.G) using the calcium phosphate transfection method at a mass ratio of 9 µg : 6 µg : 3 µg per 100-mm dish. Viral supernatants were harvested at 48 and 72 h post-transfection, concentrated by centrifugation (3,234 × g, 30 min, 4 °C), and used to transduce target HSC6 cells (for *in vitro* assays) and MTCQ1 cells (for *in vivo* tumor models) in the presence of 8 µg/mL polybrene. After 12 h of transduction, the medium was replaced, and stable clones were selected with 1 µg/mL puromycin for 5 days. The knockdown efficiency was rigorously validated by Western blotting prior to downstream functional assays.

### Western blotting

The cells and tissues were lysed in RIPA buffer supplemented with protease and nuclease inhibitors and then centrifuged at 12 000 × g for 15 min at 4 °C. The resulting supernatant was collected, and the protein concentration was quantified via a bicinchoninic acid assay. Equal amounts of protein were separated by 10% SDS–PAGE and transferred onto PVDF membranes. After blocking with 5% (w/v) skim milk, the membranes were incubated with primary antibodies against Bax (Proteintech, Cat# 50599-2-Ig), Bcl2 (Proteintech, Cat# 12789-1-AP), Caspase-3 (Proteintech, Cat# 19677-1-AP), SR-BI (ABclonal, Cat# A1584) and β-actin (Proteintech, Cat# 20536-1-AP) overnight at 4 °C with gentle agitation. Following incubation with appropriate horseradish peroxidase (HRP)-conjugated secondary antibodies, immunoreactive bands were visualized via a chemiluminescence imaging system (Bio-Rad ChemiDoc XRS+). Band intensities were quantified with ImageJ software.

### CCK-8 assay

The cells were seeded in 96-well plates at a density of 3 000 cells per well and cultured overnight. The medium was then replaced with fresh medium containing the test compounds at various concentrations (0, 2, 5, 10, and 20 µM), with an equal volume of DMSO included as a vehicle control. Each condition was performed in quadruplicate. After 24 h of treatment, 10 µL of Cell Counting Kit-8 (CCK-8; Beyotime, Cat# C0037) reagent was added to each well, and the plates were incubated at 37 °C for 2 h in the dark. The absorbance was measured at 450 nm via a microplate reader. The blank control (medium alone) value was subtracted, and the cell viability was normalized to that of the untreated control and expressed as a percentage. All the experiments included six technical replicates and were independently repeated at least three times.

### Colony-forming assay

For the colony-forming assay, cells were plated at a low density of 1 000 cells per well in 6-well plates and cultured in complete DMEM supplemented with 10% (v/v) FBS, 1% (v/v) L-glutamine, and 2% (v/v) penicillin‒streptomycin. The cultures were maintained for 7 days at 37 °C under 5% CO₂, and the medium was replaced every 3 days. Following incubation, the cells were fixed with 4% paraformaldehyde at room temperature and stained with 0.5% (w/v) crystal violet in methanol. The plates were thoroughly rinsed with distilled water and air-dried prior to imaging. Colonies were defined as clusters containing ≥ 50 adherent cells. The total number of colonies per well was recorded. The data represent the means ± standard deviations from three independent experiments.

### Migration and invasion assays

Cell migration and invasion assays were performed using 24-well Transwell chambers with 8.0 μm pore polycarbonate membranes. Prior to seeding, the cells were serum starved overnight, harvested, and resuspended in serum-free medium. A total of 50 000 cells in 200 µL of serum-free medium were seeded into the upper chamber. The lower chamber was filled with 600 µL of complete medium supplemented with 10% FBS to serve as a chemoattractant. The chamber assembly was incubated at 37 °C under 5% CO₂ for 24 h. Following incubation, nonmigrated cells on the upper membrane surface were removed via a cotton swab. The migrated cells on the lower surface were fixed with 4% paraformaldehyde, stained with 0.1% crystal violet, and imaged under a light microscope. Five randomly selected fields per membrane were counted at 100× magnification. All the experiments were performed in triplicate and were repeated three independent times.

### Wound healing assay

The cells were seeded in 12-well plates and grown to 90–100% confluence. A sterile 200 µL pipette tip was used to create a straight scratch. The detached cells were removed by washing with PBS. Fresh medium containing the designated treatments was added. Images of the same wound location were captured at 0 and 12 h under a microscope. The relative wound closure rate was calculated as follows: (initial wound area - final wound area)/initial wound area × 100%.

### Mito-tracker staining

The cells were stained with 200 nM MitoTracker probe (Yeasen, Cat# 40741ES50) and incubated for 30 min under standard culture conditions. Following incubation, the cells were washed twice with phosphate-buffered saline (PBS) and maintained in complete culture medium prior to imaging. The mitochondrial structures were visualized via superresolution structured illumination microscopy (HIS-SIM). Images were acquired with a 100×/1.50 NA oil immersion objective (Olympus).

### Immunostaining

Cells were incubated with MitoTracker probe as mentioned above, fixed with 3% formaldehyde for 30 min, blocked with 10% BSA for 30 min, permeabilized with 0.2% (v/v) Triton X-100 solution for 10 min, and incubated overnight with primary antibodies against ceramide (Enzo Life Sciences, Cat# ALX-804-196), followed by anti-mouse Alexa Fluor™ 488 secondary antibody (Thermo Fisher Scientific, Cat# A-21202) incubation. Images were detected by HIS-SIM and analyzed via ImageJ.

### Seahorse

The OCR and ECAR were assessed via an XF96 extracellular flux analyzer (Seahorse Bioscience) with an XF Cell Mitochondrial Stress Test Kit (Agilent, Cat# 103015-100) and an XF Glycolysis Stress Test Kit (Agilent, Cat# 103020-100), respectively. Data were acquired and analyzed via Wave software (version 2.2.0). The cells were seeded in XF96 plates at a density of 10 000 cells per well and allowed to adhere overnight. Mitochondrial respiratory parameters were evaluated by the sequential addition of 1.5 µM oligomycin (ATP synthase inhibitor), 2 µM carbonyl cyanide-p-trifluoromethoxyphenylhydrazone (FCCP; uncoupler), and a combination of 0.5 µM rotenone (complex I inhibitor) plus 0.5 µM antimycin A (complex III inhibitor). Glycolytic function was profiled under basal conditions and following the administration of 10 mM glucose, 1 µM oligomycin, and 50 mM 2-deoxy-D-glucose (2-DG; a glycolytic inhibitor). All measurements were normalized to the cell count per well and conducted in biological replicates.

### Flow cytometry analysis

For apoptosis detection, the cells were resuspended in 100 µL of Annexin V binding buffer (BioLegend, Cat# 640945). 1 µL of Annexin V-FITC (BioLegend, Cat# 640945) was added, and the cells were incubated for 1 h at 4 °C in the dark. Following incubation, 1 µL of 7-AAD (BioLegend, Cat# 420404) was added, and the samples were further incubated for 5 min at room temperature in the dark. The cells were then diluted with 200 µL of Annexin V binding buffer and analyzed immediately via flow cytometry (Cytoflex, Beckman Coulter, USA).

For the JC-1 mitochondrial membrane potential assay, the cells were stained with 2 µM JC-1 (Beyotime, Cat# C2003S) at 37 °C for 20 min and then washed twice with PBS. Fluorescence was measured by flow cytometry (Cytoflex, Beckman Coulter, USA) via the FITC (monomers) and PE (aggregates) channels. The ratio of aggregate to monomer fluorescence was used to quantify the ΔΨm.

For the calcein AM cobalt quenching assay, cells were loaded with 1 µM calcein AM (Beyotime, Cat# C2009S) and 1 mM CoCl₂ for 15 min at 37 °C. Ionomycin (2 µM) served as a positive control. The fluorescence intensity was measured via flow cytometry (FITC channel) (Cytoflex, Beckman Coulter, USA).

### Statistical analysis

The data are presented as the means ± SDs unless otherwise specified. Group comparisons were performed via Student’s t test or one-way or two-way ANOVA with appropriate post hoc tests, as appropriate. The MR analysis significance was set at *p* < 0.05. Statistical analyses were performed via GraphPad Prism 9.0 and R 4.4.1.

## Results

### Depression increases head and neck cancer risk and promotes oral squamous cell carcinoma progression

**Fig. 1:**
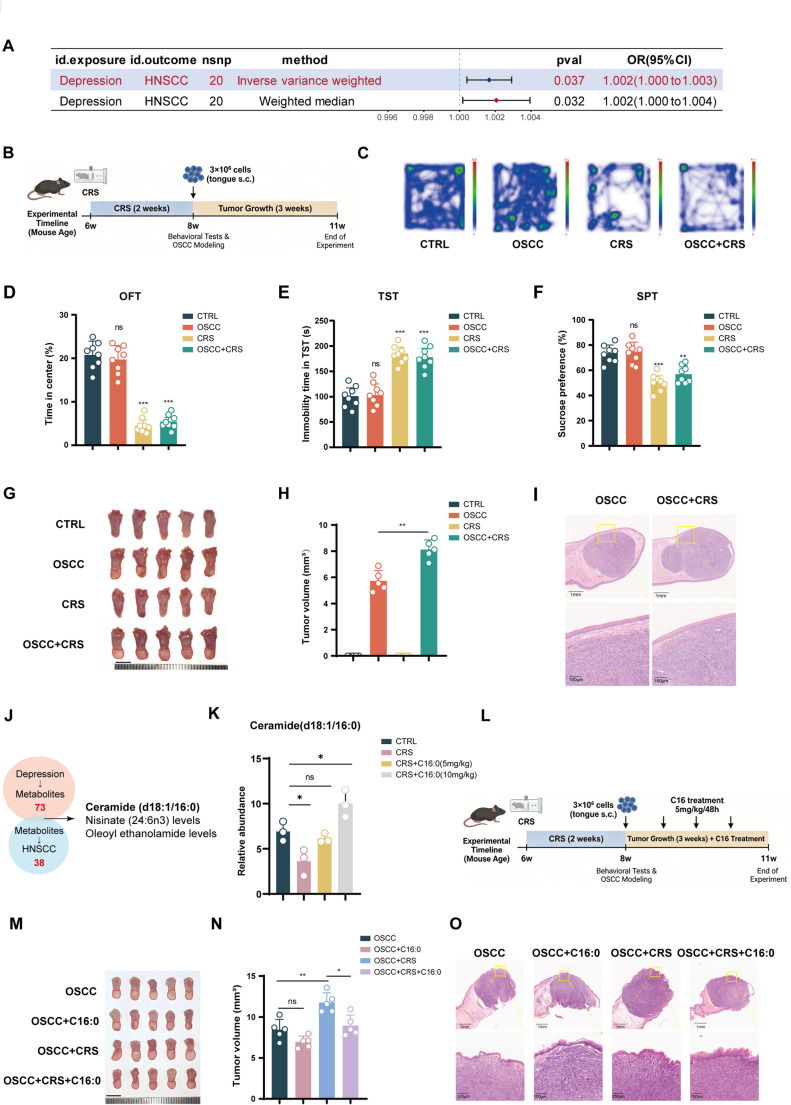
Depression promotes OSCC progression via systemic C16-ceramide depletion. **A** Causal estimates from the Mendelian randomization (MR) analysis of depression on HNSCC risk, analyzed by the Inverse Variance Weighted (IVW) and Weighted Median methods. **B** Schematic of the chronic restraint stress (CRS) and orthotopic tongue tumor mouse model. **C** Representative heatmaps of mouse movement trajectories in the OFT. **D** Time spent in the center zone in the OFT (n = 8 mice per group). **E** Immobility time in TST (n = 8 mice per group). **F** Sucrose preference percentage in SPT (n = 8 mice per group). **G** Representative photographs of tongue tumors from each group of mice (n = 5 mice per group, scale bar 5 mm). **H** Quantitative analysis of tongue tumor volume across the experimental group (n = 5 mice per group). I Representative Hematoxylin and Eosin (H&E) staining of tongue tumors from mice in (H)(scale bar 1 mm, 100 μm). **J** Venn diagram identifying overlapping metabolites significantly associated with both depression (from Supplementary Fig.3A) and HNSCC (from Supplementary Fig.3B). **K** Quantitative relative abundance of plasma Ceramide(d18:1/16:0) across the CTRL, CRS, CRS+C16:0 (5 mg/kg), and CRS+C16:0 (10 mg/kg) groups measured by mass spectrometry-based lipidomics (n = 3 per group). **L** Schematic of the mouse model for exogenous C16‑ceramide supplementation. **M** Representative photographs of tongue tumors from each group of mice(n = 5 mice per group, scale bar 5 mm). **N** Quantitative analysis of tongue tumor volume across the experimental groups (n = 5 mice per group). **O** Representative H&E stained sections of tongue tumors from mice in (N)(scale bar 1 mm, 100 μm). Data in A are presented as odds ratios (OR) with 95% confidence intervals. Data in D, E, F, H, K and N are presented as mean ± SD. Statistical significance was determined by one-way ANOVA followed by appropriate post-hoc tests. ns, not significant; *p < 0.05, **p < 0.01, ***p < 0.001.

To assess whether depression causally influences HNSCC risk, we conducted Mendelian randomization analysis. Genetic instruments were derived from large-scale genome-wide association studies of depression. We selected independent single-nucleotide polymorphisms (SNPs) associated with depression at genome-wide significance (*p* < 5 × 10⁻⁸), applying clumping to exclude SNPs in linkage disequilibrium (r² < 0.001 within 10 000 kb). All instruments showed strong statistical power (F statistic > 10), minimizing weak instrument bias. Using the inverse-variance weighted (IVW) and the weighted median methods, we found evidence that depression increased the risk of HNSCC (Fig. 1A and Supplementary Table S1). Sensitivity analyses revealed no significant horizontal pleiotropy or heterogeneity (Supplementary Fig. 1A), supporting the robustness of the association. Leave-one-out analysis (Supplementary Fig. 1B) and a forest plot of individual SNP effects (Supplementary Fig. 1C) confirmed that the overall estimate was not driven by any single genetic variant.

Given that OSCC constitutes the largest anatomical subgroup of HNSCC and shares core pathogenic pathways, we next focused on OSCC to validate and mechanistically dissect this association in vivo and in vitro. To examine whether depression promotes OSCC in vivo, we established a mouse model combining chronic restraint stress (CRS) with an orthotopic tongue cancer model (Fig. 1B). CRS mice displayed clear depression-like behaviors across multiple tests. In the open field test (OFT), they spent less time in the center (Fig. 1C, D) and traveled a significantly shorter total distance (Supplementary Fig. 2A). To confirm that this reduced locomotion was due to psychomotor retardation rather than physical motor impairment, we performed grip strength tests. The muscle strength of the stressed mice remained completely normal (Supplementary Fig. 2B). To further rule out the effects of physical restraint on motor capacity, we evaluated the free-moving chronic unpredictable mild stress (CUMS) model. The CUMS mice exhibited the same phenotype: a reduced total distance in the OFT with unchanged grip strength (Supplementary Fig. 2C and D). This proves that the decreased locomotion reflects a depressive-like state rather than a physical motor deficit. Consistently, CRS mice remained immobile longer in the tail suspension test **(**Fig. 1E**)** and showed reduced sucrose preference in the sucrose preference test **(**Fig. 1F**)**. In an orthotopic tongue cancer model, CRS significantly promoted OSCC tumor growth compared to the control group **(**Fig. 1G, H**)**. H&E staining further confirmed the malignant histopathological features of the resulting tumors **(**Fig. 1I**)**.

### Circulating C16‑ceramide acts as a key metabolic mediator of depression‑driven OSCC progression

To investigate the metabolic mechanisms linking depression and HNSCC, we conducted MR analysis on circulating metabolites. The results revealed that depression significantly altered the levels of multiple circulating metabolites (Supplementary Fig. 3A and Supplementary Table S2). Concurrently, the analysis identified several circulating metabolites causally associated with HNSCC risk (Supplementary Fig. 3B and Supplementary Table S3). By intersecting the findings from these two MR analyses, we identified three metabolites that were significant in both the “depression → metabolite” and “metabolite → HNSCC” causal pathways (Fig. 1J and Supplementary Fig. 3C). Based on literature evidence, we selected C16‑ceramide, a lipid with established roles in stress response and tumor progression, for subsequent functional validation. Its protective association in the MR analyses suggested a potential negative mediating role in the process by which depression promotes HNSCC.

To verify the depletion of C16-ceramide in depression, we performed lipidomic profiling on plasma samples from the CTRL and CRS mice. The results showed that chronic stress induced systemic lipid changes, including a broad decrease in sphingolipids (Supplementary Fig. 4A). Notably, plasma C16-ceramide levels dropped to 52.3% of the control baseline (Fig. 1K). Furthermore, total ceramide levels, the direct precursor C16-dihydroceramide [Cer(d18:0/16:0)], and other major ceramide species were also significantly decreased (Supplementary Fig. 4B-E). Exogenous C16-ceramide supplementation restored plasma C16-ceramide to near or above baseline levels (Fig. 1K). However, it did not reverse the global lipid changes caused by CRS, such as the increased lysophosphatidylcholines (LPCs) and decreased fatty acids (FAs) (Supplementary Fig. 4A). This indicates that C16-ceramide acts as a specific downstream mediator, rather than a general treatment for the systemic stress response.

The CRS model involves physical restraint. To rule out the effects of immobility or altered feeding behaviors, we performed plasma lipidomic analysis using the CUMS model, which allows free movement (Supplementary Fig. 4F). Although some FAs and LPCs showed different trends between the two models, C16-ceramide and other major ceramide species were consistently decreased in the CUMS model (Supplementary Fig. 4G and K). These results from both models confirm that the decrease in circulating ceramides is driven by the depressive-like state itself, rather than physical inactivity.

To validate the functional relevance of this metabolite specifically in the oral cavity, the primary site of HNSCC, we performed exogenous C16‑ceramide supplementation in a mouse model combining CRS and orthotopic tongue cancer (Fig. 1L). We selected a dose of 5 mg/kg, which successfully restored plasma C16-ceramide to the normal baseline without causing the lipid overload seen at higher doses (10 mg/kg) (Fig. 1K). Importantly, supplementation with C16-ceramide partially reversed the tumor-promoting effect of depression. This conclusion was supported by measurements of tumor volume (Fig. 1M, N) and histopathological analysis (Fig. 1O).

#### C16-Ceramide suppresses malignant phenotypes of OSCC cells

**Fig. 2:**
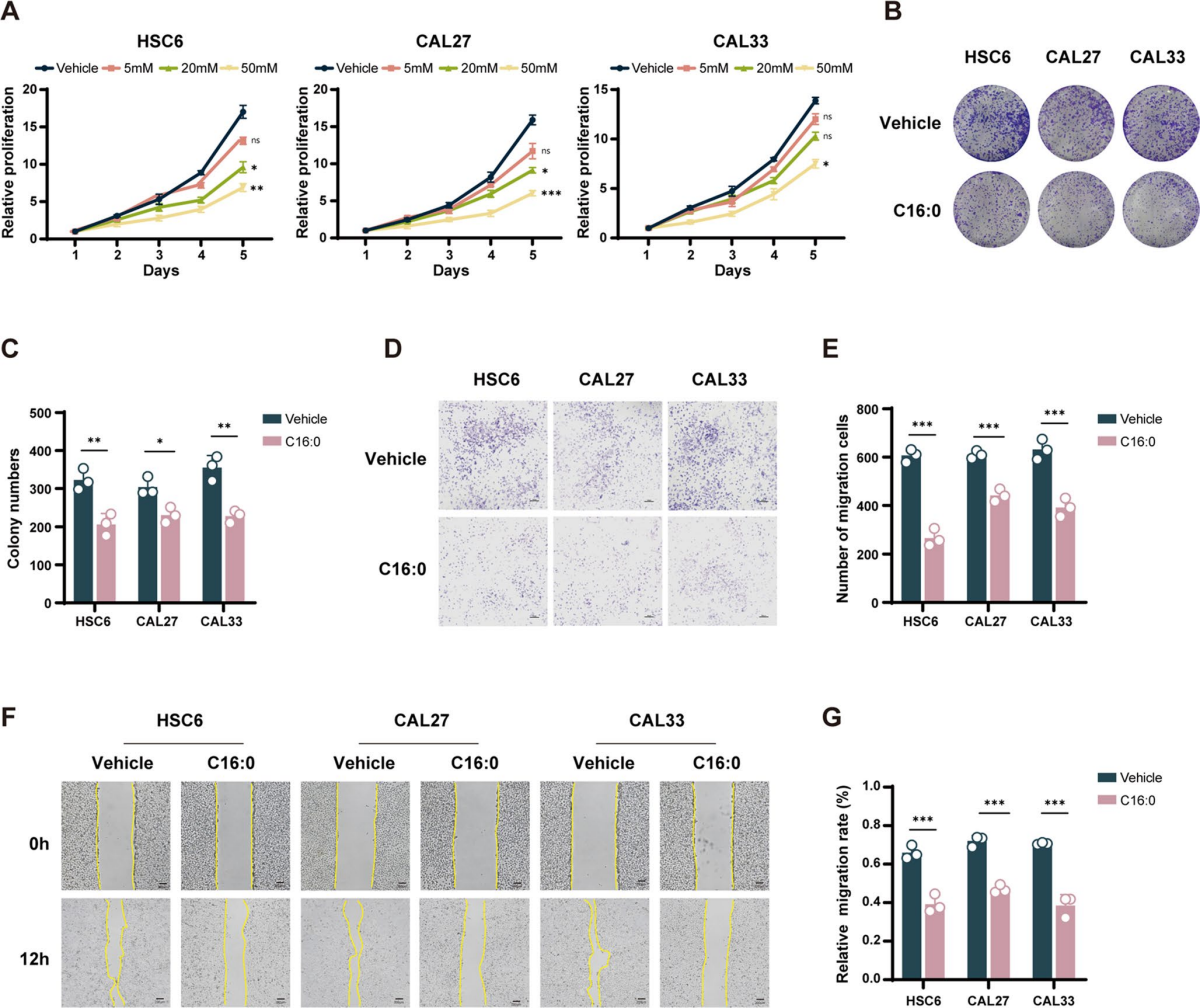
C16-ceramide suppresses malignant phenotypes of OSCC cells. **A** Proliferation of OSCC cell lines (HSC6, CAL27, CAL33) treated with indicated concentrations of C16-ceramide (C16:0) or vehicle control, measured by CCK-8 assay over time. **B** Representative images of colonies formed by OSCC cells after treatment with C16-ceramide or vehicle control in colony formation assays. **C** Quantitative statistical analysis of the colony formation assays shown in (B). **D** Representative images from transwell migration assays of OSCC cells treated with C16-ceramide or vehicle control (scale bar 100 μm). **E** Quantitative statistical analysis of the transwell migration assays shown in (D). **F** Representative images from wound healing (scratch) assays of OSCC cell monolayers treated with C16-ceramide or vehicle control (scale bar 200 μm). **G** Quantitative statistical analysis of the wound healing assays shown in (F), measuring the relative wound closure over time. Data in A, C, E, and G are presented as mean ± SD from at least three independent experiments. Statistical significance was determined by two-way ANOVA (for A) or one-way ANOVA (for C, E and G) followed by appropriate post-hoc tests. ns, not significant; *p < 0.05, **p < 0.01, ***p < 0.001

To investigate the direct effects of C16‑ceramide on oral squamous cell carcinoma (OSCC) cells, we performed a series of functional assays in multiple human HSC6 cell lines (HSC6, CAL27 and CAL33). The CCK‑8 assay showed that C16‑ceramide inhibited cell proliferation in a concentration‑dependent manner **(**Fig. 2A**)**. Colony formation assays further demonstrated a significant reduction in the number of cell colonies following C16‑ceramide treatment **(**Fig. 2B, C**)**. In transwell migration assays, fewer cells passed through the membrane in the C16‑ceramide‑treated group compared to the control, indicating a marked suppression of migratory capacity **(**Fig. 2D, E**)**. Wound‑healing assays also confirmed that C16‑ceramide treatment significantly delayed scratch closure and reduced the relative wound closure rate **(**Fig. 2F, G**)**. Together, these functional assays demonstrated that C16-ceramide inhibits the proliferation, clonogenicity and migration of OSCC cells.

### SCARB1 is specifically highly expressed in OSCC epithelial cells and facilitates intracellular accumulation of C16-ceramide

**Fig. 3:**
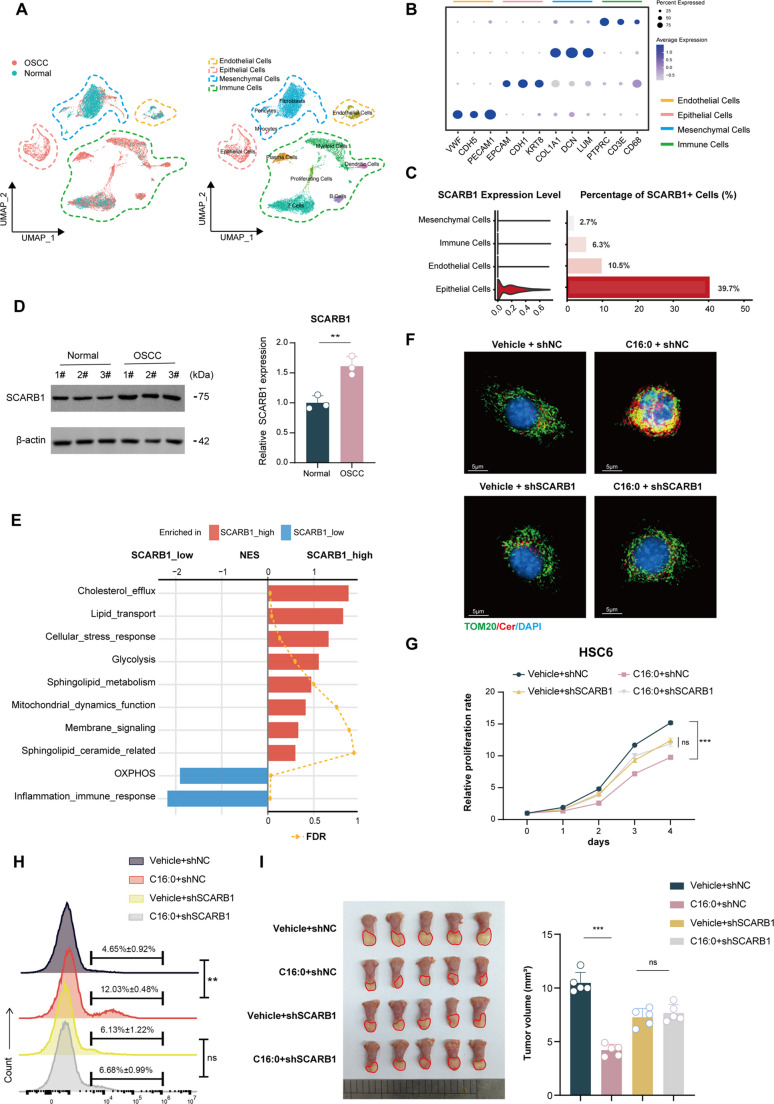
SCARB1 mediates C16-ceramide uptake, and its knockdown abolishes C16-ceramide-induced tumor suppression**. A** Uniform Manifold Approximation and Projection (UMAP) plots of single‑cell RNA‑seq data from oral tissues. Left: cells colored by tissue type (Normal vs. OSCC). Right: cells colored by major cell‑type clusters (Epithelial, Immune, Endothelial, and Mesenchymal cells). **B** Bubble plot displaying canonical marker genes for each cell-type cluster. **C** Left: Violin plots showing the expression level of SCARB1 across the four major cell-type clusters. Right: Bar graph showing the percentage of SCARB1-positive cells within each cluster. **D** Representative Western blot images (left) and corresponding densitometric quantification of SCARB1 protein expression (right) in normal tongue tissues versus OSCC tongue tissues from mice. β-Actin was used as an internal loading control. **E** Gene-set enrichment analysis (GSEA) scores for custom-defined pathways in SCARB1-high versus SCARB1-low tumor epithelial cells. **F **Representative immunofluorescence images demonstrating the co-localization of ceramide (Cer, red) and mitochondria (TOM20, green) in HSC6 cells across the four treatment groups. Nuclei were counterstained with DAPI (blue) (scale bar 5 μm). **G** Relative proliferation rate measured by CCK-8 assay across the four treatment groups. **H** Representative flow cytometry histograms and quantitative analysis of apoptosis rates in HSC6 cells across the corresponding groups. **I** Representative photographs of orthotopic tongue tumors (left) and quantitative analysis of tumor volume (right) across the four in vivo groups. Data are presented as mean ± SD from at least three independent experiments. Statistical significance was determined by unpaired Student’s t-test (for D), two-way ANOVA (for G), or one-way ANOVA (for H and I) followed by appropriate post-hoc tests. ns, not significant; **p < 0.01, ***p < 0.001

To elucidate the mechanism by which C16-ceramide directly acts on tumor cells, we analyzed single-cell transcriptomic data from normal oral tissues and OSCC tissues obtained from public databases. After batch-effect correction, dimensionality reduction and clustering, we identified 11 clusters, which were categorized into four major types: epithelial, endothelial, mesenchymal and immune cells (Fig. 3A). Characteristic marker genes for each cluster are displayed in a bubble plot (Fig. 3B and Supplementary Fig. 5A). Given that high-density lipoprotein is known to systemically transport C16-ceramide, we focused on the expression pattern of its receptor, scavenger receptor class B member 1 (SCARB1). The analysis revealed that *SCARB1* was specifically highly expressed in epithelial cells (Fig. 3C and Supplementary Fig. 5B). Western blotting further confirmed that SCARB1 protein levels were significantly higher in orthotopic tongue tumor tissues than in normal tongue tissues in mice (Fig. 3D).

To gain functional insight into SCARB1-high epithelial cells, we divided them into SCARB1-high and SCARB1-low groups and performed gene set enrichment analysis. The results showed that SCARB1-high cells were significantly enriched in pathways related to lipid transport, cholesterol efflux and cellular stress response (Fig. 3E), suggesting that these cells possess active lipid-handling capacity and increased stress sensitivity, potentially rendering them more responsive to circulating C16-ceramide. To functionally confirm that SCARB1 acts as the indispensable conduit for C16-ceramide uptake, we established a stable SCARB1-knockdown HSC6 OSCC cell line (shSCARB1) (Supplementary Fig. 6A). Co-localization immunofluorescence revealed that while exogenous C16-ceramide robustly accumulated in the mitochondria of control cells (shNC), this intracellular entry and subsequent mitochondrial targeting were largely abolished following SCARB1 knockdown (Fig. 3F). This demonstrates that C16-ceramide effectively enters OSCC epithelial cells and targets the mitochondrial compartment via a SCARB1-dependent mechanism.

Functionally, the anti-proliferative and pro-apoptotic effects of C16-ceramide were completely neutralized in shSCARB1 cells (Fig. 3G, H). Similarly, the inhibitory effects of C16-ceramide on colony formation, cell migration, and invasion were entirely eliminated (Supplementary Fig. 6B-D). Crucially, we sought to validate this indispensable role of SCARB1 in vivo. Using the orthotopic tongue cancer model in CRS mice, we confirmed the knockdown of SCARB1 in the tumor tissues by Western blotting (Supplementary Fig. 6E). Consistent with our in vitro functional data, while systemic C16-ceramide supplementation significantly mitigated tumor growth in mice bearing shNC tumors, it completely failed to exert any anti-tumor efficacy in mice bearing shSCARB1 tumors (Fig. 3I and Supplementary Fig. 6F). Together, these data provide definitive in vitro and in vivo evidence that SCARB1 directly mediates the intracellular uptake of circulating C16-ceramide, which in turn triggers mitochondrial apoptosis.

Interestingly, our in vitro and in vivo rescue experiments revealed a nuanced metabolic phenomenon: knockdown of SCARB1 alone partially suppressed baseline OSCC proliferation and tumor growth **(**Fig. 3G, I**)**. This is likely because SCARB1 classically acts as a key transporter for nutritional lipids (such as HDL-cholesterol), which rapidly proliferating tumor cells heavily rely on for membrane biogenesis. Therefore, SCARB1 depletion induces a cytostatic “lipid starvation” state that stunts cell growth, yet simultaneously protects the cells from the toxic, apoptosis-inducing C16-ceramide pathway.

### C16-ceramide induces mitochondrial apoptosis via mitochondrial dysfunction and metabolic reprogramming

**Fig. 4:**
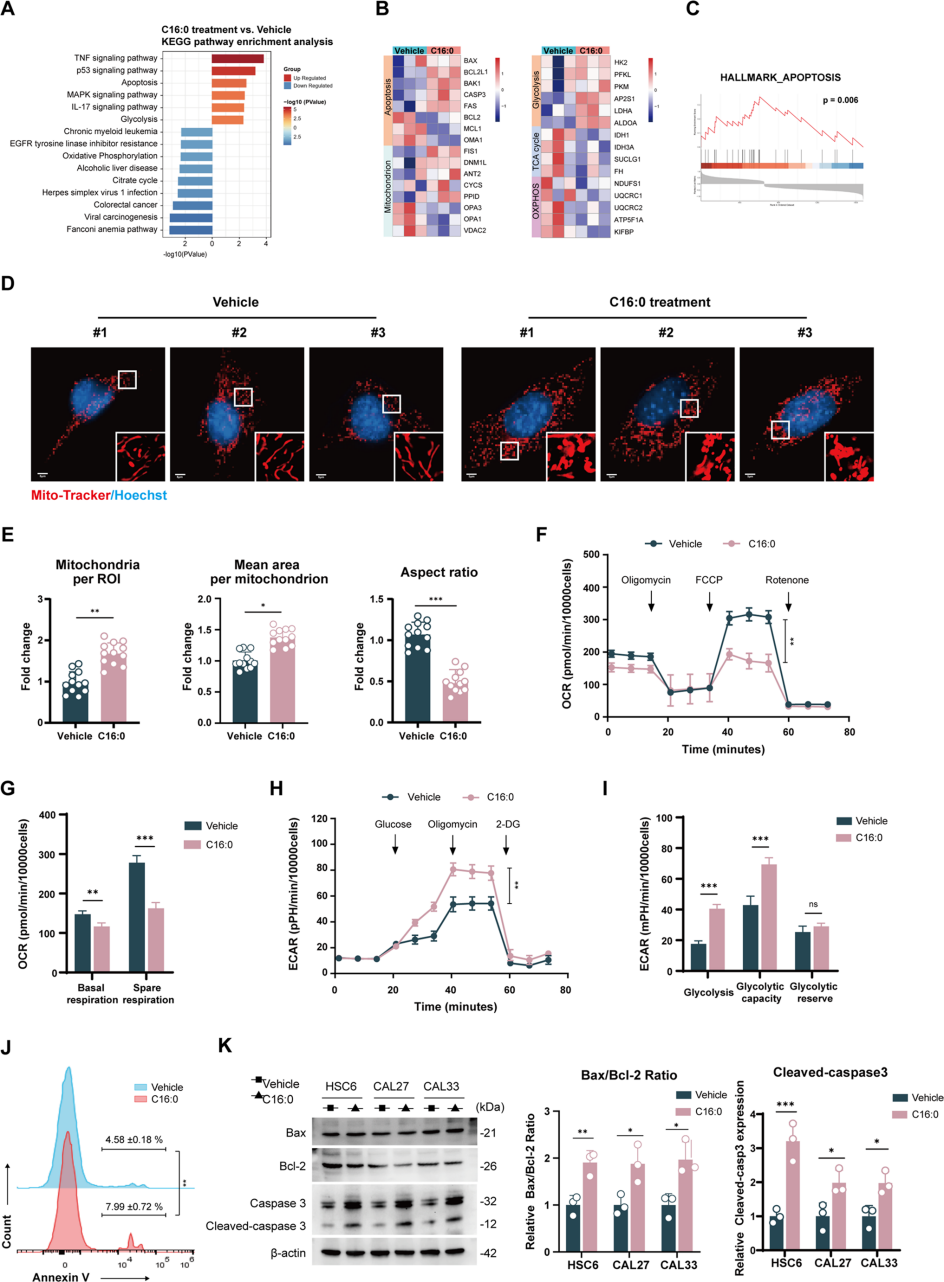
C16-ceramide induces mitochondrial dysfunction and metabolic reprogramming to trigger apoptosis in OSCC cells. **A** Kyoto Encyclopedia of Genes and Genomes (KEGG) pathway enrichment analysis of differentially expressed genes between C16-ceramide (C16:0) and vehicle-treated OSCC cells. **B** Heatmap showing the expression patterns of key differentially expressed genes involved in apoptosis, mitochondrial function, glycolysis, the tricarboxylic acid (TCA) cycle, and oxidative phosphorylation (OXPHOS). **C** Gene Set Enrichment Analysis (GSEA) plot showing significant enrichment of an apoptosis-related gene signature in C16-ceramide–treated cells. **D** Representative immunofluorescence images of mitochondria (MitoTracker, red) and nuclei (Hoechst, blue) in vehicle- and C16-ceramide–treated cells (scale bar 5 μm). **E** Quantitative analysis of mitochondrial morphology, including mitochondrial density (number per region of interest), mean mitochondrial area, and aspect ratio. F Real-time oxygen consumption rate (OCR) measured by Seahorse XF Analyzer. **G** Quantitative analysis of basal respiration and spare respiratory capacity derived from OCR measurements. **H** Real-time extracellular acidification rate (ECAR) measured by Seahorse XF Analyzer. **I** Quantitative analysis of glycolytic parameters, including glycolysis, glycolytic capacity, and glycolytic reserve derived from ECAR measurements. **J** Quantification of Annexin V–positive apoptotic cells. **K** Representative Western blot images (left) of key apoptosis-related proteins in OSCC cell lines treated with vehicle or C16-ceramide. The corresponding bar graph displays the densitometric quantification of the relative Bax/Bcl-2 protein ratio (middle) and Cleaved-caspase3 expression (right). β-Actin was used as an internal loading control. Data in E, G, I, J and K are presented as mean ± SD from at least three independent experiments. Statistical significance was determined by unpaired two-tailed Student’s t-test (for E, G, I, J and K) or two-way ANOVA tests (for F and H). ns, not significant; *p < 0.05, **p < 0.01, ***p < 0.001

To determine how C16-ceramide exerts its effects, we performed RNA sequencing on control and C16-ceramide–treated OSCC cells. KEGG enrichment analysis revealed that differentially expressed genes were significantly enriched in pathways related to apoptosis, glycolysis and oxidative phosphorylation **(**Fig. 4A**)**. A heatmap further revealed expression changes in genes involved in apoptosis, mitochondrial function, glycolysis, the TCA cycle and oxidative phosphorylation **(**Fig. 4B**)**. Gene set enrichment analysis (GSEA) confirmed significant activation of the apoptosis pathway **(**Fig. 4C**)**. Morphologically, C16-ceramide treatment induced severe mitochondrial abnormalities **(**Fig. 4D**)**, characterized by increased mitochondrial density, a larger mean area, and a reduced aspect ratio, suggesting mitochondrial swelling and fragmentation **(**Fig. 4E**)**. Seahorse energy metabolism analysis indicated that C16-ceramide significantly decreased the oxygen consumption rate (OCR), impairing basal respiration and sparing respiratory capacity **(**Fig. 4F, G**)**. Concurrently, the extracellular acidification rate (ECAR) increased, and the glycolytic capacity was increased, indicating typical metabolic reprogramming **(**Fig. 4H, I**)**. Flow cytometry revealed that C16-ceramide treatment significantly increased the percentage of apoptotic cells **(**Fig. 4J**)**. Western blot analysis further confirmed that C16-ceramide upregulated the expression of the pro-apoptotic proteins Bax and Cleaved Caspase-3 while downregulating the anti-apoptotic protein Bcl-2 **(**Fig. 4K**)**. These results demonstrate that C16-ceramide triggers mitochondrial dysfunction, leading to the initiation of the intrinsic apoptotic pathway.

### C16-ceramide triggers mitochondrial apoptosis via mPTP opening

**Fig. 5:**
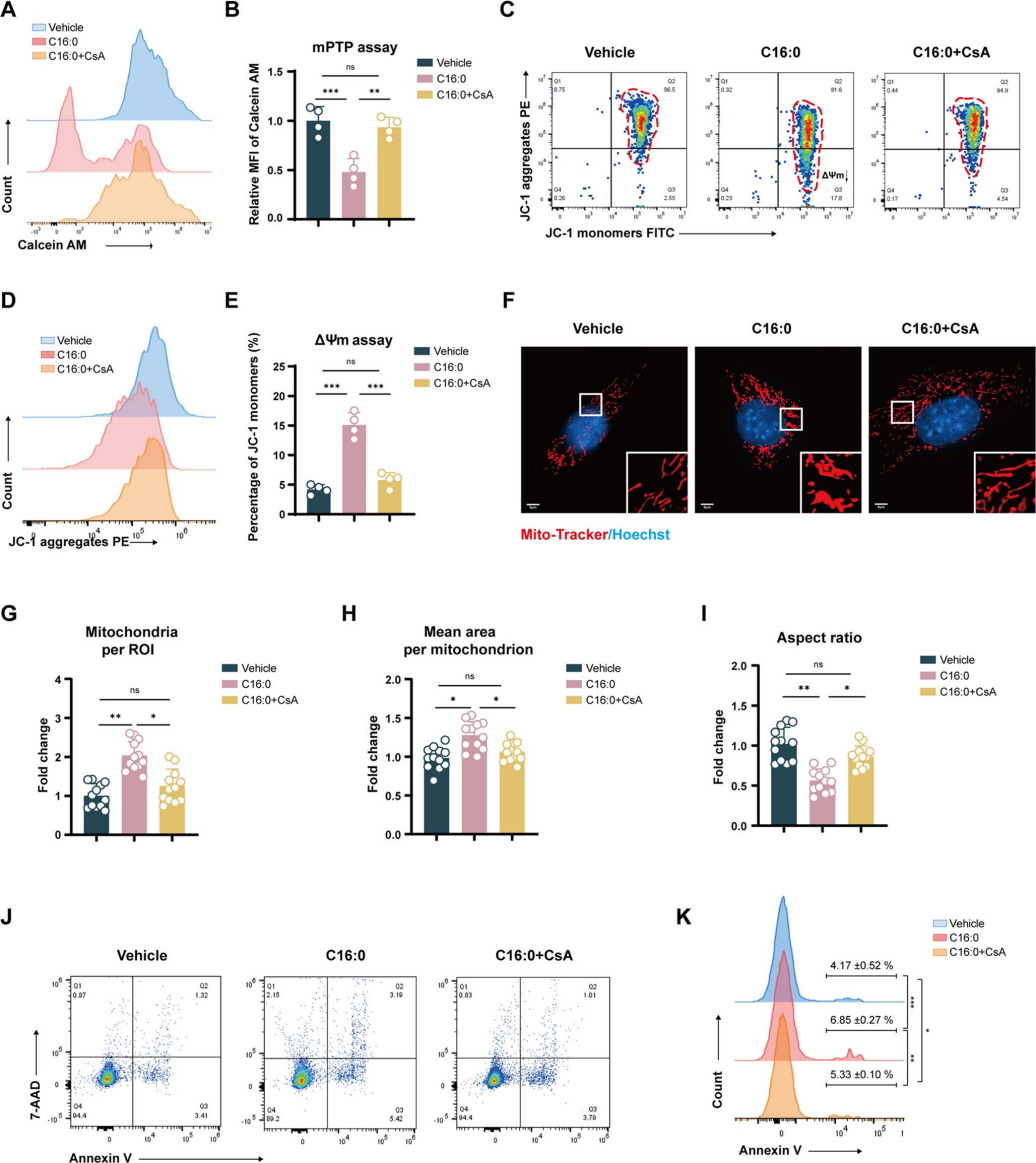
C16-ceramide triggers the mitochondrial apoptosis pathway by opening mPTP. **A** Flow cytometry histograms of the Calcein AM cobalt quenching assay. The fluorescence intensity of Calcein AM (green) is quenched by CoCl₂ upon mPTP opening, and ionomycin serves as a positive control for maximum quenching. **B** Quantitative analysis of the Calcein AM cobalt quenching assay shown in (A). The relative mean fluorescence intensity (MFI) of Calcein AM (in the presence of CoCl₂) inversely indicates the degree of mPTP opening. **C** Representative flow cytometry dot plots of JC-1 staining. The shift from JC-1 aggregates (PE, high ΔΨm) to monomers (FITC, low ΔΨm) indicates mitochondrial membrane potential (ΔΨm) dissipation. **D** Flow cytometry histograms of JC-1 monomers (FITC channel) from the experiment shown in (C). **E** Quantitative analysis of the percentage of cells with depolarized mitochondria (JC-1 monomers) from (C) and (D). **F** Representative immunofluorescence images of mitochondria (stained with MitoTracker, red) and nuclei (stained with Hoechst, blue) in cells treated with vehicle, C16-ceramide (C16:0), or C16-ceramide plus the mPTP inhibitor Cyclosporin A (CsA) (scale bar 5 μm). **G** Quantitative analysis of mitochondrial density (number per region of interest). **H** Quantitative analysis of the mean area per mitochondrion. **I** Quantitative analysis of mitochondrial aspect ratio. **J** Representative flow cytometry dot plots of Annexin V/7-AAD staining for apoptosis detection in cells under the indicated treatments. **K** Quantitative histogram showing the percentage of Annexin V-positive apoptotic cells. Data in B, E, G, H, I, and K are presented as mean ± SD from at least three independent experiments. Statistical significance was determined by one-way ANOVA (for B, E, G, H, I and K) followed by appropriate post-hoc tests for multiple comparisons.ns, not significant; *p < 0.05; **p < 0.01; ***p < 0.001.

We next asked how C16-ceramide induces mitochondrial dysfunction. Using the calcein AM cobalt quenching assay, we observed a marked decrease in fluorescence upon C16-ceramide treatment, indicating increased opening of the mPTP **(**Fig. 5A, B**)**. JC-1 staining further confirmed the loss of the mitochondrial membrane potential (ΔΨm), as shown by a shift from JC-1 aggregates to monomers **(**Fig. 5C-E**)**. Crucially, the mPTP-specific inhibitor cyclosporin A (CsA) reversed C16-ceramide-induced mitochondrial abnormalities **(**Fig. 5F**)**. Pretreatment with CsA restored mitochondrial morphology, reducing the density and mean area while increasing the aspect ratio **(**Fig. 5G-I**)**. CsA also strongly attenuated C16-ceramide–induced apoptosis **(**Fig. 5J, K**)**. Together, these data establish mPTP opening as the key event in C16-ceramide–triggered mitochondrial apoptosis.

### The C16-Ceramide–mPTP axis suppresses tumor progression and is clinically relevant

**Fig. 6:**
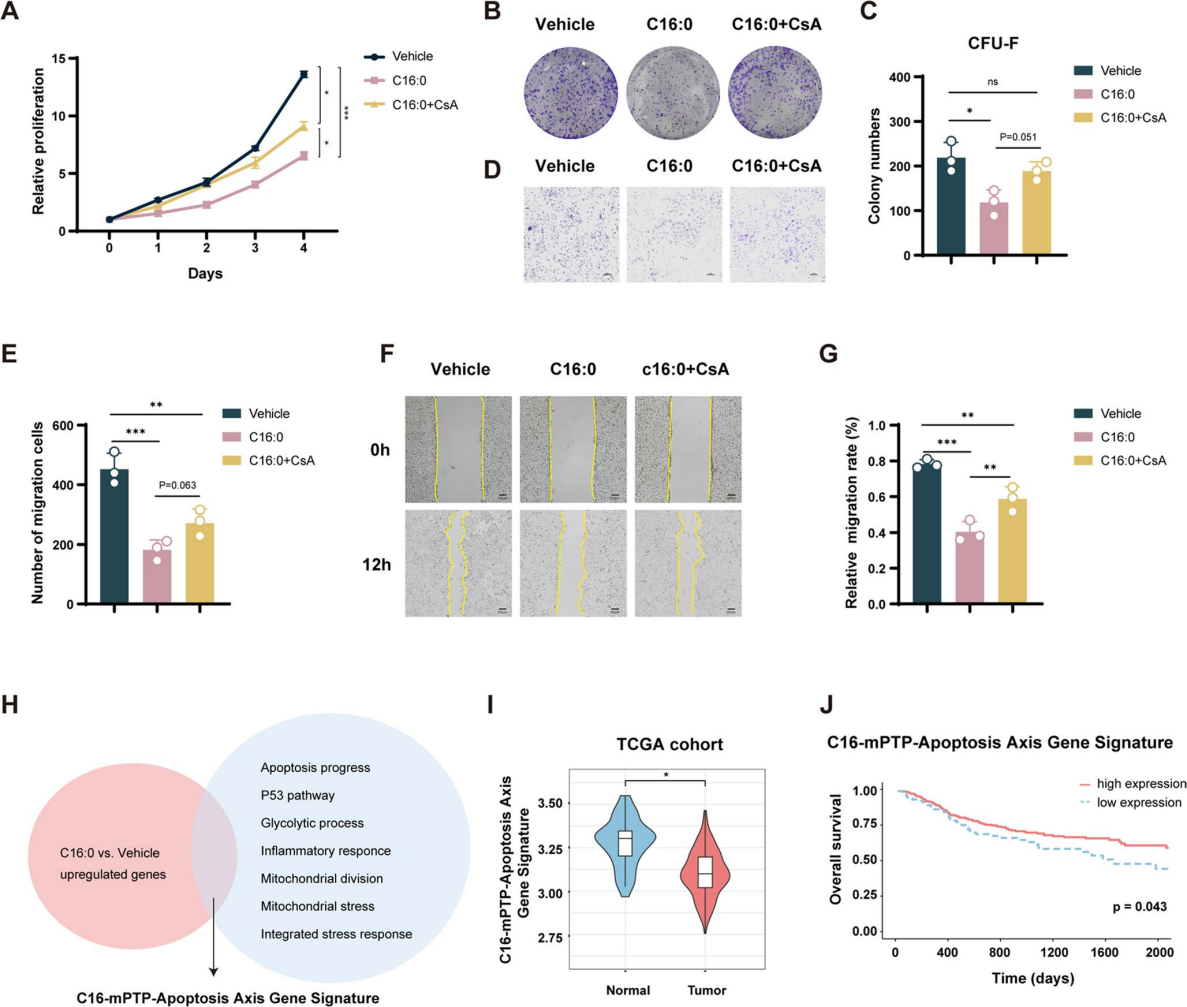
The C16-ceramide-mPTP axis plays a critical role in OSCC progression. **A** Cell proliferation assessed by CCK-8 assay in OSCC cells treated with vehicle, C16-ceramide (C16:0), or C16-ceramide plus Cyclosporin A (C16:0 + CsA) over time. **B** Representative images of colony formation (CFU-F assay) in OSCC cells under the indicated treatments. **C** Quantitative statistical analysis of the colony formation assays shown in (B). **D** Representative images from transwell migration assays of OSCC cells under the indicated treatments (scale bar 100 μm). **E** Quantitative statistical analysis of the transwell migration assays shown in (D). **F** Representative images from wound healing (scratch) assays ofOSCC cell monolayers under the indicated treatments at 0 and 12 h (scale bar 200 μm). **G** Quantitative statistical analysis of the relative wound closure rate from the assays shown in (F). **H** Venn diagram identifying the overlapping upregulated genes which constitute the “C16-mPTP-Apoptosis Axis Gene Signature”. **I** Expression levels of the “C16-mPTP-Apoptosis Axis Gene Signature” in normal versus tumor tissues from The Cancer Genome Atlas (TCGA) OSCC cohort. **J** Kaplan-Meier survival curve analysis based on the “C16-mPTP-Apoptosis Axis Gene Signature” in the TCGA OSCC cohort. Data in A, C, E, and G are presented as mean ± SD (or SEM) from at least three independent experiments.Statistical significance was determined by an unpaired t-test (for I), one-way ANOVA (for C, E and G) or two-way ANOVA (for A) followed by appropriate post-hoc tests for multiple comparisons.ns, not significant; *p < 0.05; **p < 0.01; ***p < 0.001

Rescue experiments confirmed that the antitumor effects of C16-ceramide depend on mPTP activation. CsA pretreatment restored proliferation **(**Fig. 6A**)**, colony formation **(**Fig. 6B, C**)**, migration **(**Fig. 6D, E**)** and wound closure **(**Fig. 6F, G**)** in C16-ceramide–treated OSCC cells. To assess clinical relevance, we constructed a “C16-mPTP-apoptosis” gene signature **(**Fig. 6H**)**. This signature was significantly downregulated in tumor samples from the TCGA OSCC cohort **(**Fig. 6I**)**. Patients with high signature expression also experienced longer overall survival (*p* = 0.029) **(**Fig. 6J**)**, underscoring the potential prognostic value of this pathway in OSCC.

## Discussion

Epidemiological evidence has long suggested a link between depression and increased cancer risk or poorer prognosis [8–10, 32, 33]. This connection is often explained by chronic activation of the hypothalamic–pituitary–adrenal (HPA) axis, which elevates glucocorticoid levels, promotes sympathetic nervous system activity, and fosters an immunosuppressive, inflammatory tumor microenvironment [11, 12, 34–36]. However, much of the supporting data remain observational, leaving the underlying molecular mechanisms incompletely resolved. Our study strengthens the causal basis of this relationship by applying Mendelian randomization, which provides genetic evidence that depression is a risk factor for HNSCC. Given that OSCC is the largest component of HNSCC, this finding strongly implicates depression in OSCC pathogenesis. More importantly, we identified a decrease in circulating C16-ceramide as a key metabolic intermediary that helps explain how depression promotes OSCC progression.

Ceramide, a central sphingolipid metabolite, is involved in diverse cellular processes—including proliferation, apoptosis, migration, and energy homeostasis. In cancer biology, intracellular ceramide accumulation is known to trigger stress-induced apoptosis and autophagy [37], while the survival of tumors often increases via the upregulation of ceramide-degrading enzymes [24, 38, 39]. Our work extends this paradigm by highlighting the role of specific circulating ceramide species, particularly C16-ceramide, as systemic tumor-suppressive messengers. Using MR analyses based on HNSCC data, we established C16-ceramide as a metabolite causally linked to both depression and HNSCC, suggesting its potential role in the OSCC subtype. In mice, depression led to significantly lower circulating C16-ceramide, and restoring its levels reversed depression-driven tumor growth. These results suggest that a systemic deficiency in C16-ceramide could be one mechanism through which depression enables OSCC development.

Importantly, the clinical relevance of stress-induced ceramide reduction is supported by human metabolomic data. While acute stress or certain depression episodes may transiently increase some lipids, extensive analyses of human cohorts subjected to long-term physical and psychological stress reveal a shift toward a hypo-metabolic “dauer” state. This state is characterized by a profound and global depletion of plasma sphingolipid and ceramide pools [31]. This clinical reality in humans closely mirrors the significant reduction of plasma C16-ceramide observed in our chronic stress mouse models. Furthermore, our lipidomic data clearly distinguish the broad effects of chronic stress from the specific effects of C16-ceramide supplementation. Chronic stress induces a widespread systemic lipid dysregulation, establishing a tumor-promoting environment. In contrast, exogenous C16-ceramide administration does not reverse the overall systemic stress response, as the dysregulation of other lipids (such as increased LPCs and decreased FAs) remains uncorrected. Instead, it acts as a specific metabolic intervention. By restoring the circulating pool of C16-ceramide, it directly targets OSCC cells via SCARB1 to induce apoptosis, compensating for the stress-induced metabolic vulnerability without reversing the global stress state itself.

A central finding of our study lies in clarifying how C16-ceramide restrains malignant behaviors in OSCC cells. Previous studies have established the broad role of ceramide in stress signaling and apoptosis [20–22], and epidemiological studies have repeatedly linked depression to cancer prognosis [6, 7]. However, these bodies of work have largely remained separate. Our work bridges this gap by coherently linking systemic psychological stress to a specific circulating metabolic alteration and subsequently to an intratumoral mitochondrial execution pathway. We showed that C16-ceramide triggers mitochondrial dysfunction, metabolic disruption, and ultimately apoptosis, thereby curbing proliferation, colony formation, and migration. Crucially, these effects depend on opening of the mPTP. This defines a “C16-ceramide–mPTP–apoptosis” axis, which we found to be clinically relevant: a corresponding gene signature derived from this pathway correlated with patient survival in the TCGA OSCC cohort.

Moreover, our findings provide a mechanistic explanation for how circulating C16-ceramide can directly and selectively act upon tumor epithelial cells. Prior evidence indicates that high-density lipoprotein (HDL), rather than small extracellular vesicles, serves as the primary carrier for circulating ceramide [40]. Here, through single-cell transcriptomic analysis of OSCC tissues, we discovered that the HDL receptor SCARB1 is specifically highly expressed in tumor epithelial cells. This pattern positions these cells as the most sensitive targets for circulating C16-ceramide. This discovery offers a novel perspective for understanding how systemic metabolic alterations can precisely exert their effects on specific cellular populations within the tumor microenvironment.

The translational implications of our findings are twofold. On the one hand, the inverse correlation between circulating C16-ceramide and OSCC risk suggests that monitoring its levels could serve as a novel biomarker for risk stratification in OSCC patients with comorbid depression. On the other hand, our data strongly support the therapeutic potential of targeting the C16-ceramide pathway. While direct administration faces pharmacological challenges, our work validates the entire downstream axis. This opens avenues for developing small-molecule mimetics or modulators that can specifically activate this apoptotic cascade. Alternatively, strategies aimed at boosting endogenous C16-ceramide synthesis or inhibiting its degradation could also emerge as novel adjunctive therapies to counteract stress-promoted tumor growth.

While our study provides a causal and mechanistic link between depression and OSCC progression, several important questions remain. First, although we observed a significant reduction in circulating C16-ceramide under depressive conditions, the primary tissue or cellular source of this circulating lipid pool is not yet identified. It is unclear whether the decrease originates from altered hepatic synthesis [41] or changes in other peripheral tissue secretion [42]. Second, the precise biochemical pathway through which the psychological state of depression leads to lower circulating C16-ceramide levels is still undefined. Depression is a complex systemic condition; it may influence the enzymes responsible for ceramide synthesis [43, 44] or degradation [38, 45], either directly through neuroendocrine signals or indirectly via downstream inflammatory mediators. Third, at the cellular level, our data suggest SCARB1 facilitates the uptake of extracellular C16-ceramide into OSCC cells. However, the subsequent journey of this lipid to the mitochondria and the exact molecular events that connect its accumulation to mPTP opening require further investigation. Finally, translating these promising preclinical findings into clinical benefit faces hurdles. The stability, bioavailability, and targeted delivery of exogenous C16-ceramide pose significant pharmacological challenges. Future efforts might focus on developing more stable analogs, small-molecule activators of the downstream pathway, or strategies to modulate endogenous ceramide metabolism in a tissue-specific manner.

**Fig. 7:**
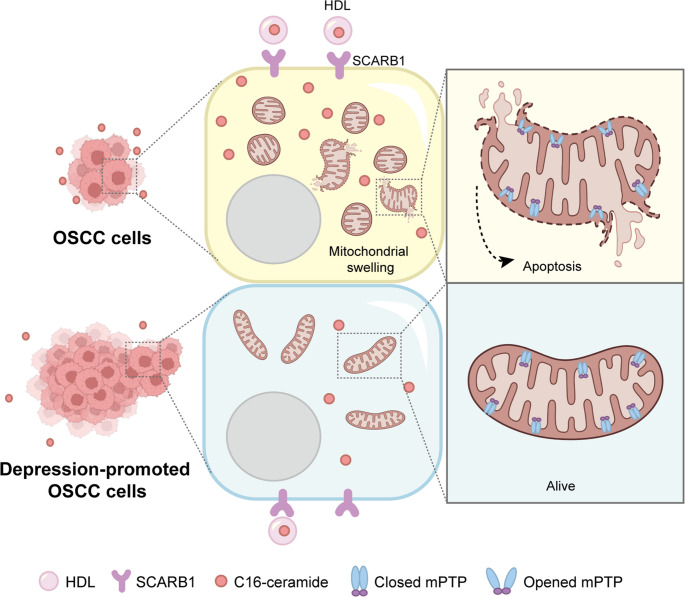
The C16-Ceramide/SCARB1/mPTP axis mediates stress-induced OSCC progression

In summary, our work reveals how depression promotes oral cancer through a specific metabolic pathway. We found that depression lowers the circulating levels of a protective lipid, C16-ceramide, which helps tumor cells avoid mitochondrial apoptosis. C16-ceramide normally suppresses tumors by opening the mPTP, causing fatal mitochondrial damage. Furthermore, we identified the potential involvement of the SCARB1 receptor in mediating the tumor-cell effect of this lipid. These results provide a mechanistic explanation linking psychological stress to cancer progression **(**Fig. 7**)**. Restoring C16-ceramide function or the activity of its related pathway may offer a new treatment strategy, especially for OSCC patients with depression.

## Supplementary Information

Below is the link to the electronic supplementary material.

Supplementary Material 1 (XLSX 31.9 KB)

Supplementary Material 2 (PDF 5.60 MB)

Supplementary Material 3 (PDF 3.63 MB)

## Data Availability

The data that support the findings of the study are available from the corresponding author upon reasonable request.
