# Supplementary material for: Depression-induced systemic C16-ceramide deficiency promotes OSCC via attenuation of mitochondrial apoptosis
Source: Cell Mol Life Sci. 2026 Jun 6;83(1):303. doi: 10.1007/s00018-026-06267-1 (PMC13469040; doi:10.1007/s00018-026-06267-1)
Supplement: Supplementary file 1 [file 18_2026_6267_MOESM2_ESM.pdf]

[SI_1.pdf]Supplementary Information:

Supplementary Figure1: Sensitivity analyses for the Mendelian randomization of depression on HNSCC risk.

Supplementary Figure2: Supplementary Behavioral assessments of the CRS and CUMS models.

Supplementary Figure3: Mendelian randomization analyses identifying circulating C16- ceramide as a mediating metabolite.

Supplementary Figure4: Plasma lipidomic profiling upon exogenous C16-ceramide supplementation and in the CUMS model.

Supplementary Figure5: Comprehensive single-cell profiling of SCARB1 expression across cellular compartments in OSCC.

Supplementary Figure6: Assessment of SCARB1 knockdown efficiency and its impact on OSCC cells in vitro and in vivo.

For the article entitled: Depression-Induced Systemic C16-Ceramide Deficiency Promotes OSCC via Attenuation of Mitochondrial Apoptosis

Submitted to: *Cellular and Molecular Life Sciences*

Authors: Yuan Pan, Jingjing Guan, Yueqi Wang, Rongchun Yang, Keyu Lai, Xijuan Chen, Juan Xia

Corresponding author: Juan Xia, Xijuan Chen

E-mail: Juan Xia (xiajuan@mail.sysu.edu.cn),

Xijuan Chen (chenxj235@mail.sysu.edu.cn)

Supplementary Figure 1

A

		Pleiotropy test			Heterogeneity test					
		MR Egger			MR Egger			Inverse variance weighted		
**id.exposure**	**id.outcome**	**Intercept**	**SE**	**pval**	**Q**	**Q_df**	**Q_pval**	**Q**	**Q_df**	**Q_pval**
Depression	HNSCC	0.0001	0.0001	0.2734	13.9767	18	0.7306	15.2529	19	0.7064

B

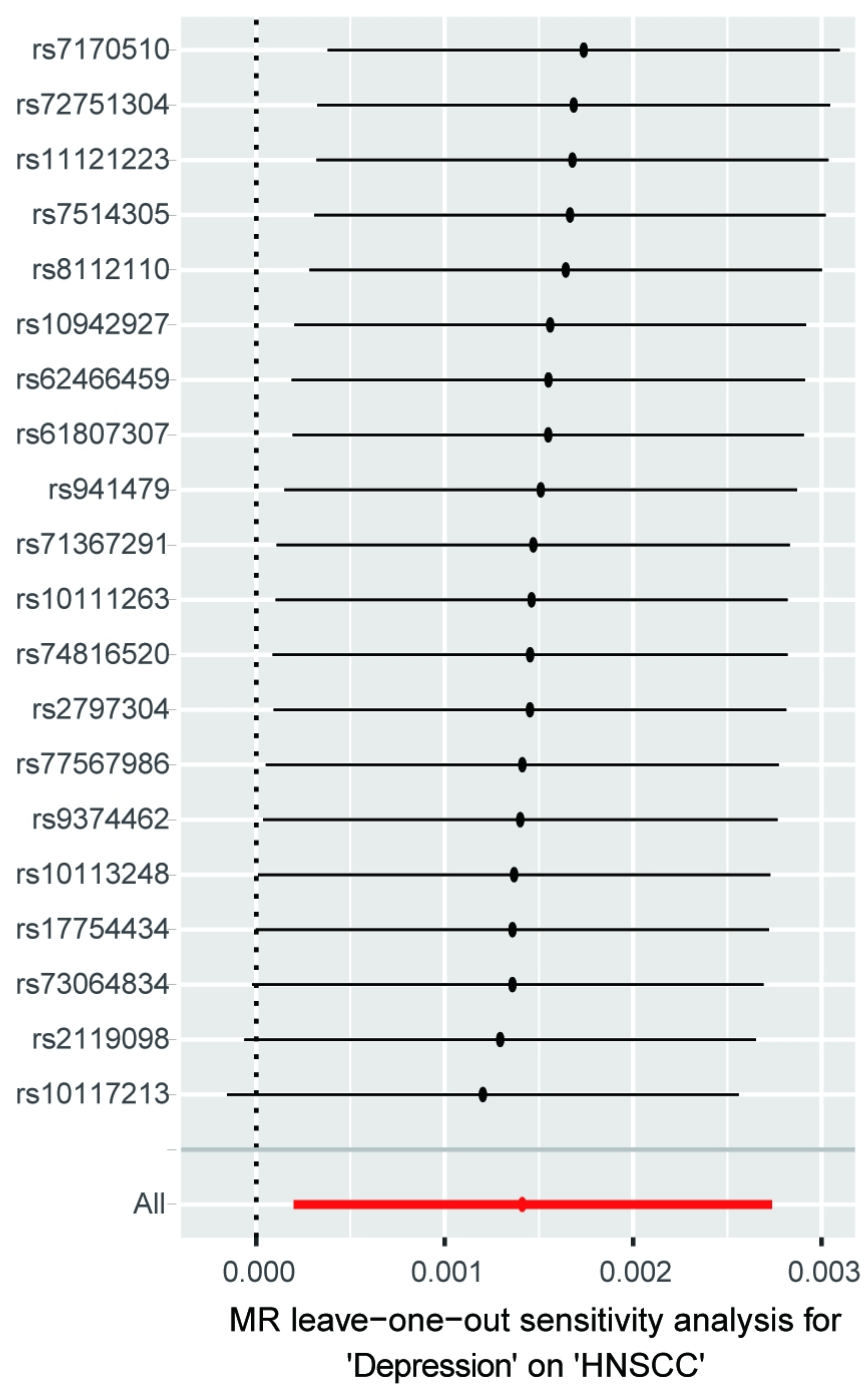

C

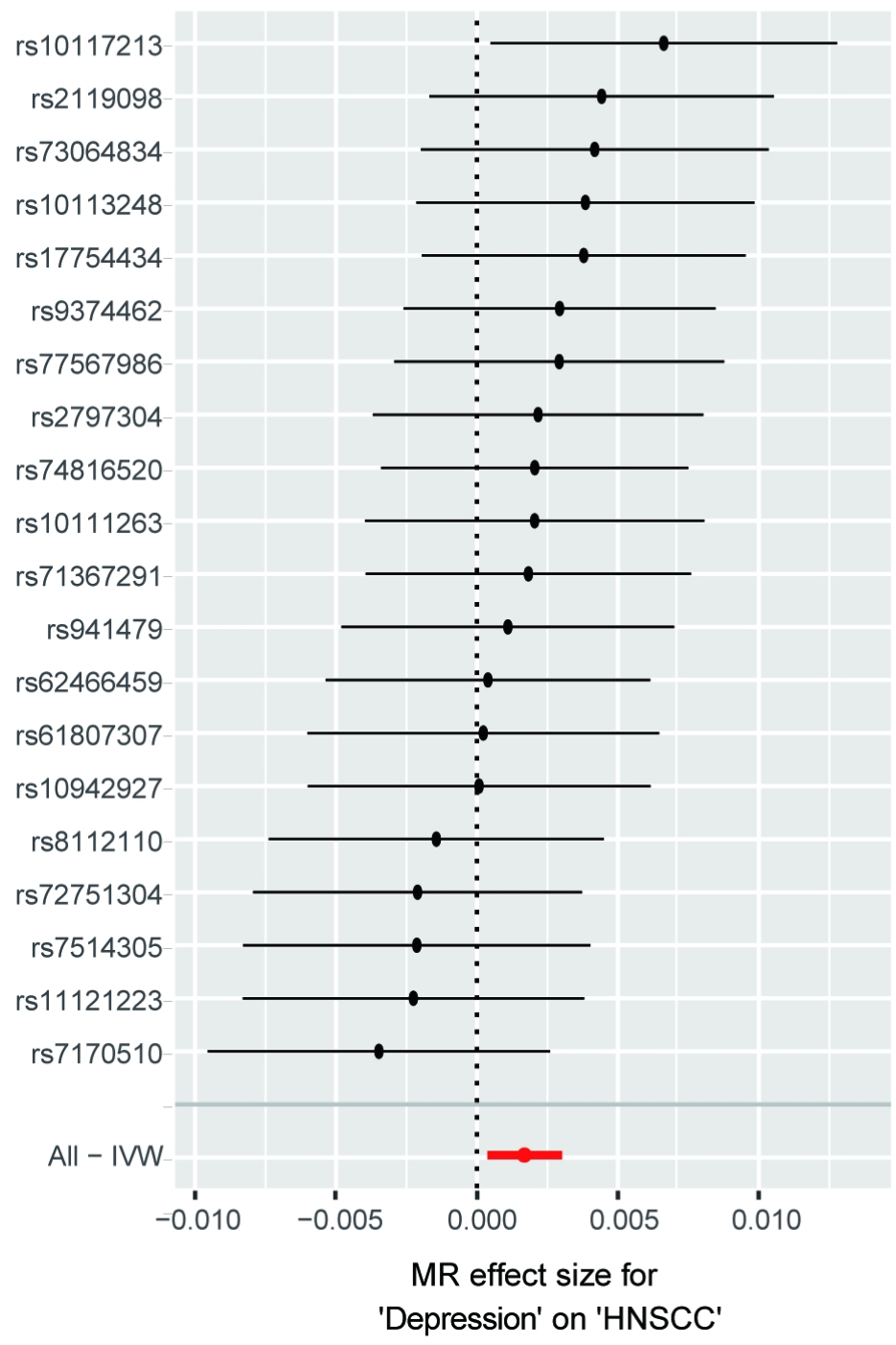

Supplementary Fig.1 Sensitivity analyses for the Mendelian randomization of depression on HNSCC risk.

A Pleiotropy and heterogeneity tests for the MR analysis of depression on HNSCC risk (corresponding to main Fig.1A). **B** Forest plot showing the causal effects of individual instrumental SNPs on HNSCC risk in the depression-to-HNSCC MR analysis. **C** Leave-one-out sensitivity analysis for the MR estimates of depression on HNSCC risk. Data in B and C are presented as odds ratios (OR) with 95% confidence intervals.

Supplementary Figure 2

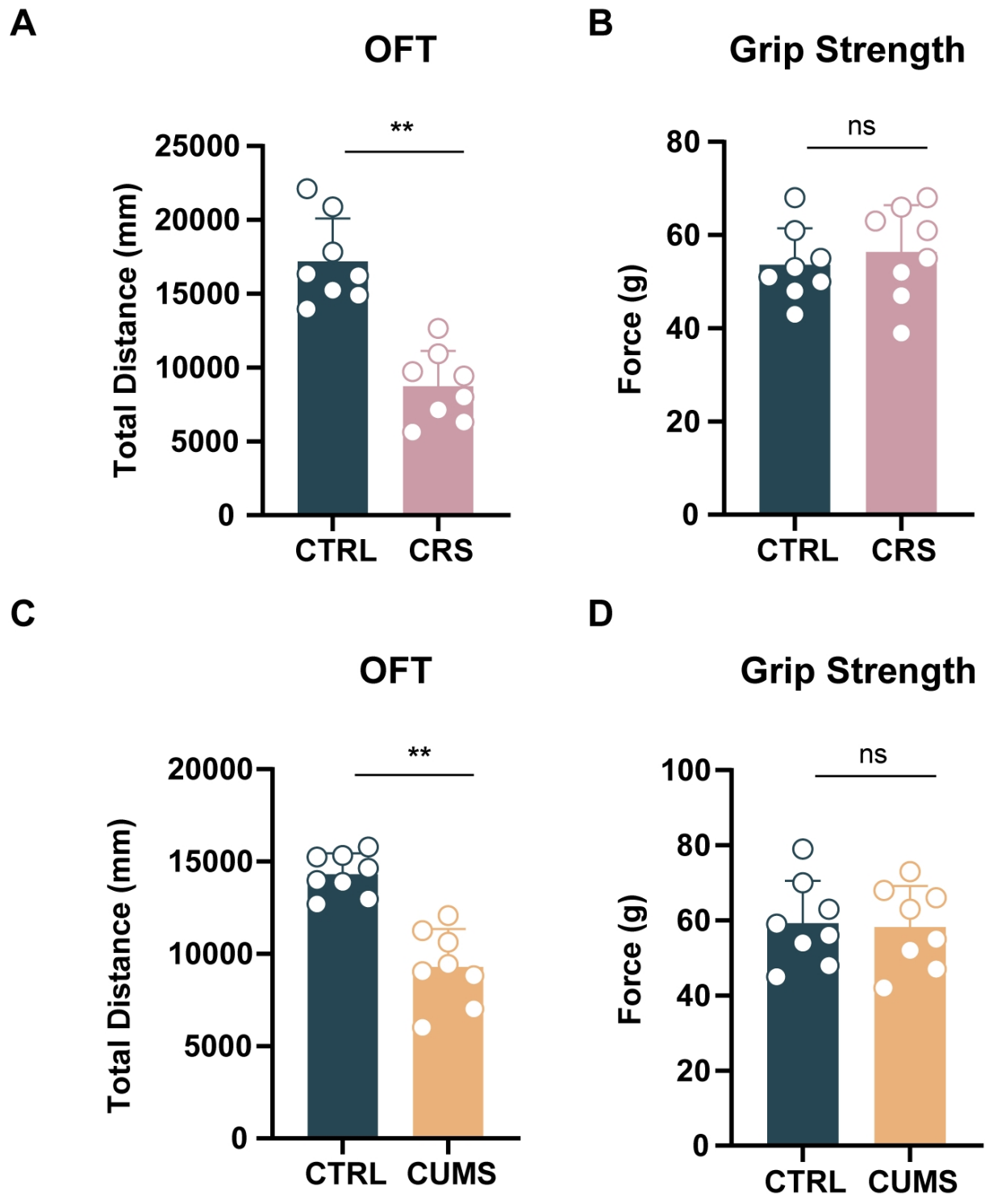

Supplementary Fig. 2 Supplementary Behavioral assessments of the CRS and CUMS models.

A Total distance traveled in the OFT in the CRS model. **B** Forelimb grip strength test results in the CRS model. **C** Total distance traveled in the OFT in the CUMS model. **D** Forelimb grip strength test results in the CUMS model. Data are presented as mean ± SD. Statistical significance was determined by unpaired two-tailed Student's t-test.

Supplementary Figure 3

A

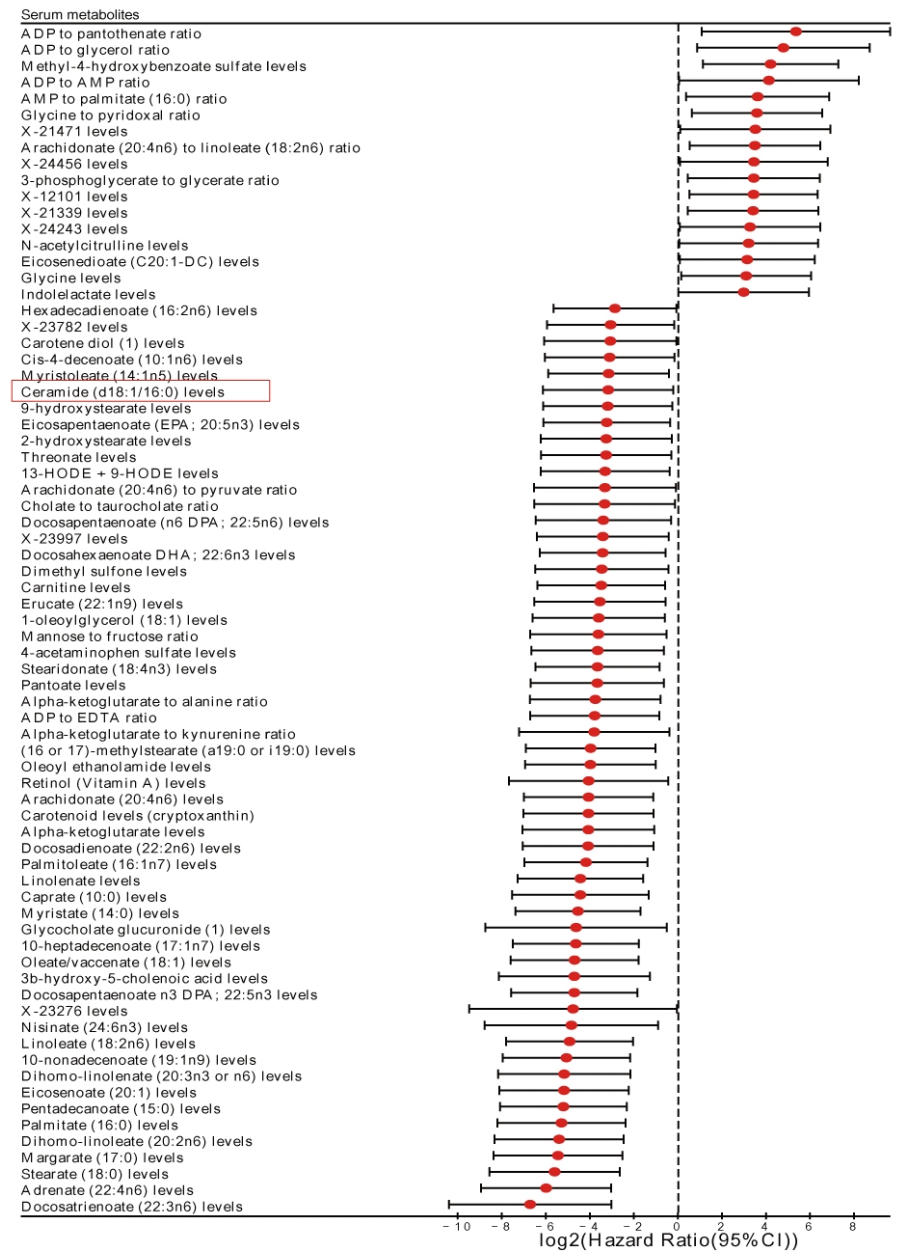

B

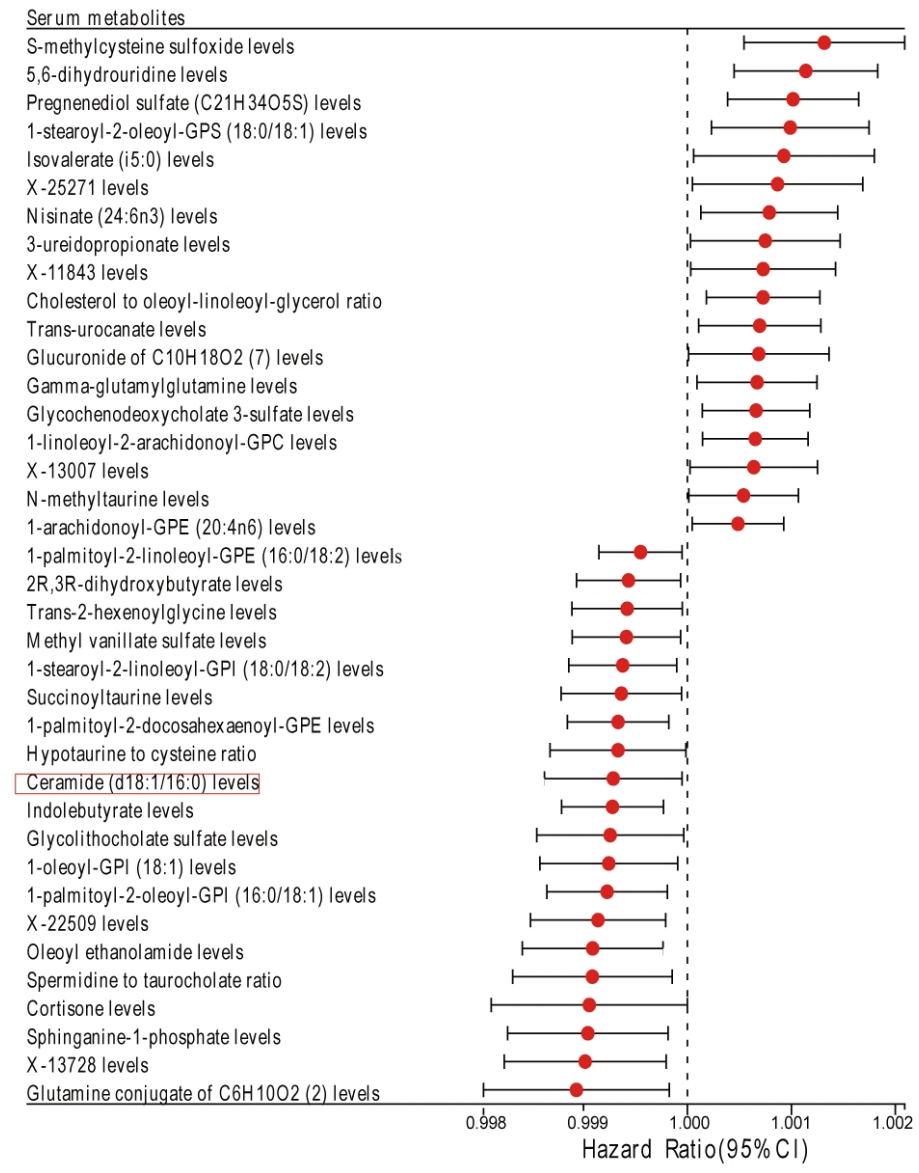

C

id.exposure	id.outcome	method	nsnp	pval	or	or_lci95	or_uci95
Depression	Ceramide (d18:1/16:0) levels	IVW	43	0.0380	0.1140	0.0147	0.8868
Depression	Oleoyl ethanolamide levels	IVW	43	0.0091	0.0650	0.0083	0.5070
Depression	Nisinate (24:6n3) levels	IVW	43	0.0168	0.0358	0.0023	0.5487
Ceramide (d18:1/16:0) levels	HNSCC	IVW	26	0.0344	0.9993	0.9986	0.9999
Oleoyl ethanolamide levels	HNSCC	IVW	24	0.0083	0.9991	0.9984	0.9998
Nisinate (24:6n3) levels	HNSCC	IVW	17	0.0191	1.0008	1.0001	1.0014

Supplementary Fig.3 Mendelian randomization analyses identifying circulating C16-ceramide as a mediating metabolite.

A MR analysis of the causal effect of depression on serum metabolite levels. Forest plot displays OR with 95% confidence intervals for significantly associated metabolites. **B** MR analysis of the causal effects of serum metabolites on HNSCC risk. Forest plot shows OR with 95% confidence intervals for significantly associated metabolites. **C** Detailed results from the inverse variance weighted (IVW) analysis for the overlapping metabolite identified in the main Fig. 1J. Data in A and B are presented as odds ratios (OR) with 95% confidence intervals.

Supplementary Figure 4

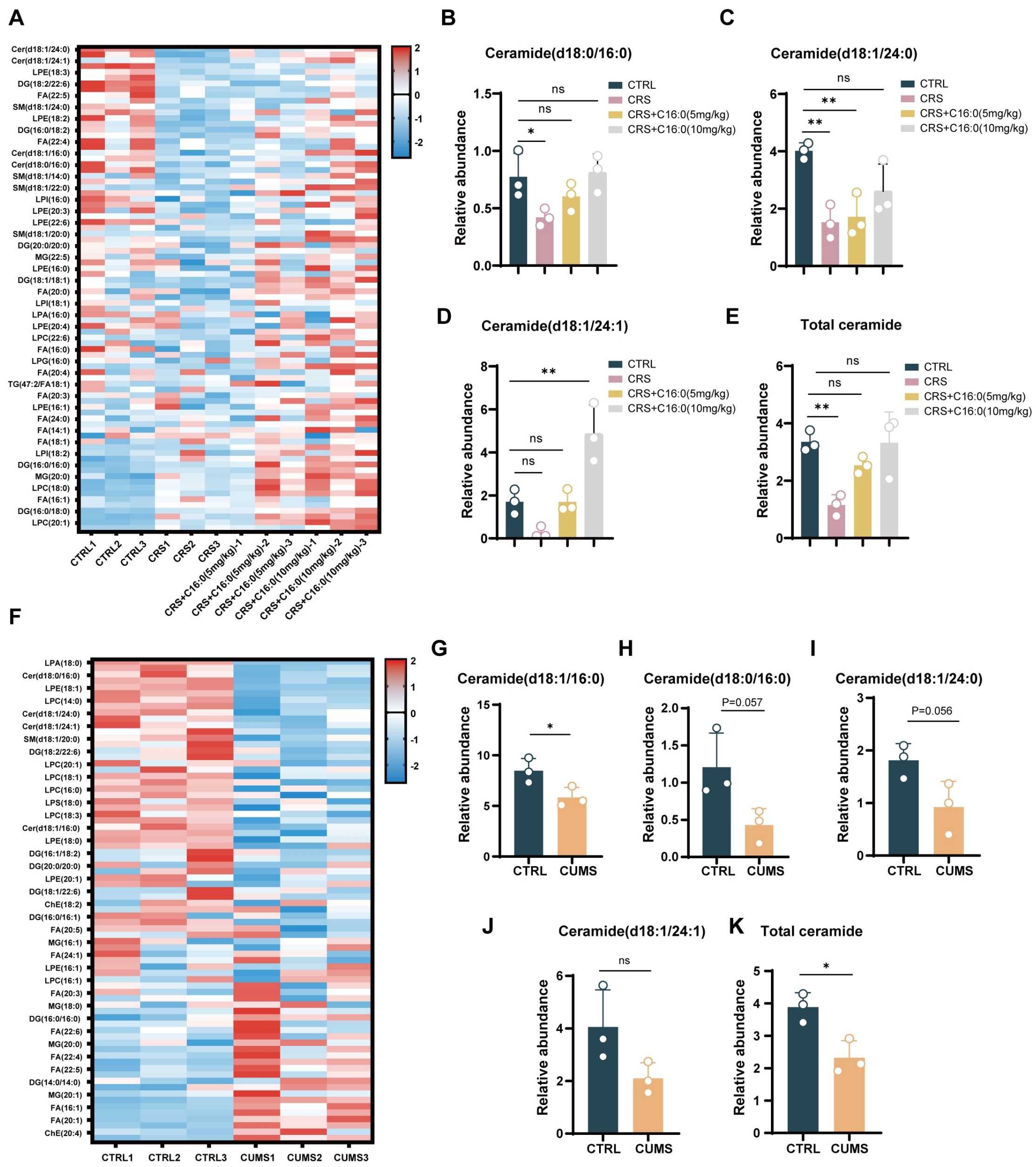

Supplementary Fig. 4 Plasma lipidomic profiling upon exogenous C16-ceramide supplementation and in the CUMS model.

A Heatmap illustrating the global plasma lipidomic alterations across the CTRL, CRS, and CRS groups treated with exogenous C16-ceramide (5 mg/kg and 10 mg/kg). **B-E** Quantitative relative abundances of Ceramide(d18:0/16:0) **(B)**, Ceramide(d18:1/24:0) **(C)**, Ceramide(d18:1/24:1) **(D)**, and total ceramide **(E)** across the four experimental groups. **F** Heatmap depicting the global plasma lipidomic profile

comparison between the CTRL and CUMS groups. **G-K** Quantitative relative abundances of Ceramide(d18:1/16:0) **(G)**, Ceramide(d18:0/16:0) **(H)**, Ceramide(d18:1/24:0) **(I)**, Ceramide(d18:1/24:1) **(J)**, and total ceramide **(K)** across the CTRL and CUMS groups. Data are presented as mean ± SD. Statistical significance was determined by one-way ANOVA (for B-E) or unpaired Student's t-test (for G-K). ns, not significant; *$p < 0.05$, **$p < 0.01$.

Supplementary Figure 5

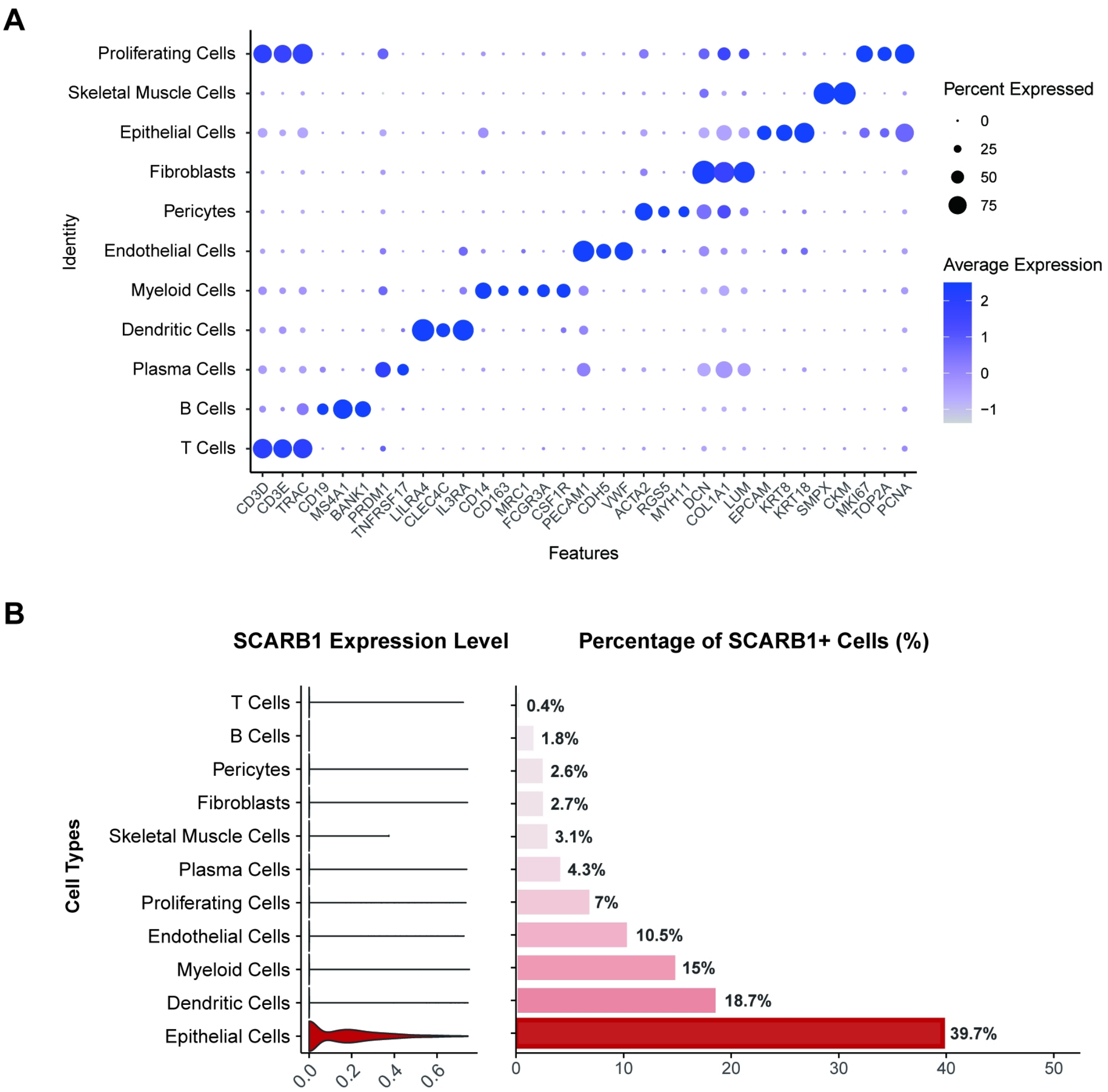

Supplementary Fig.5 Comprehensive single-cell profiling of SCARB1 expression across cellular compartments in OSCC.

A Bubble plot annotating 11 distinct cell clusters identified by single-cell RNA sequencing of OSCC tissues. **B** Left: Violin plots depicting the expression level of *SCARB1* across the 11 cell clusters. Right: Bar graph quantifying the percentage of *SCARB1*-positive cells within each of the 11 cell clusters.

Supplementary Figure 6

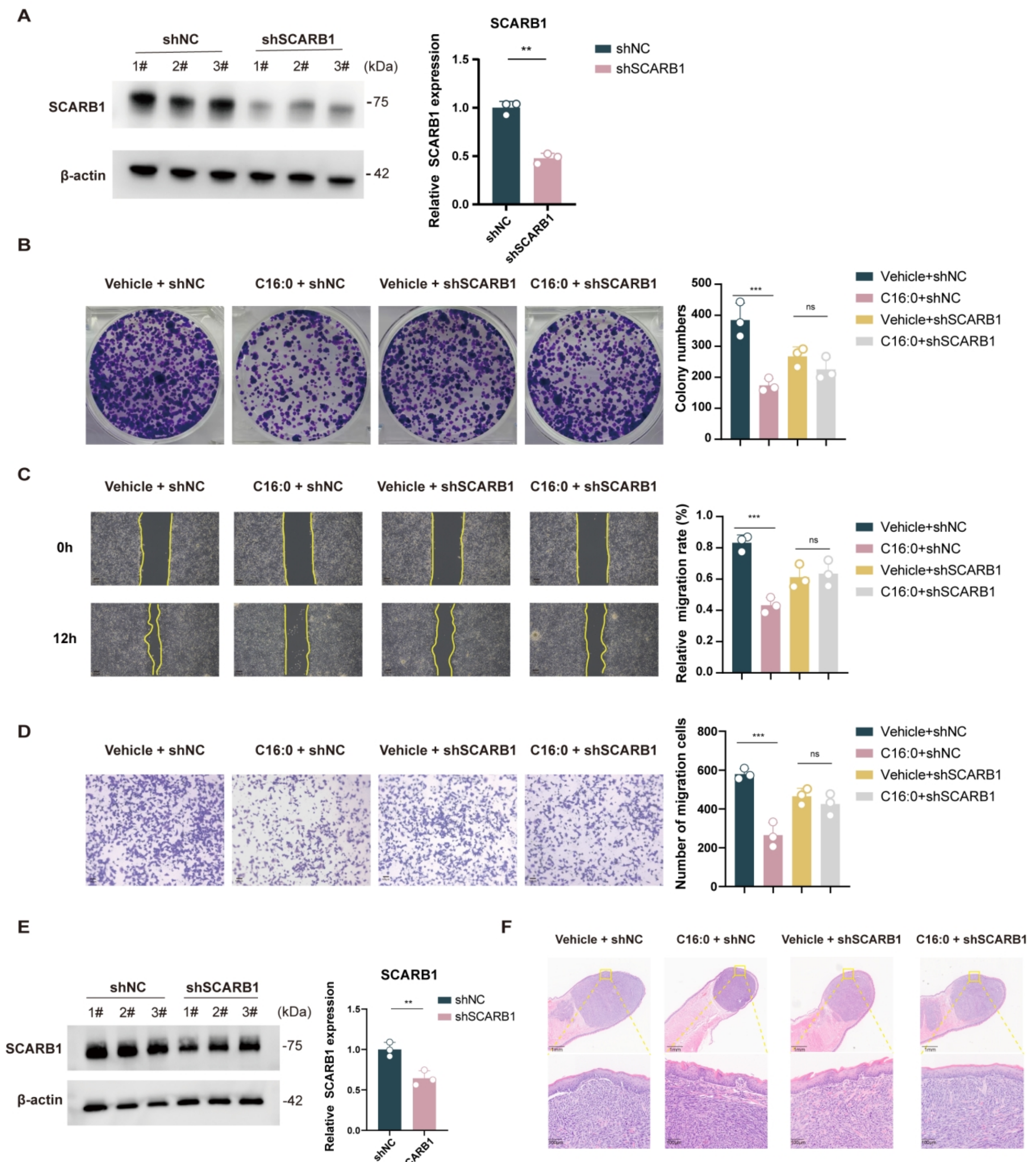

Supplementary Fig. 6 Assessment of SCARB1 knockdown efficiency and its impact on OSCC cells in vitro and in vivo.

A Representative Western blot images (left) and corresponding densitometric quantification of SCARB1 protein expression (right) in the HSC6 cell line (shNC vs. shSCARB1). **B** Representative images (left) and quantitative statistical analysis (right) of colony formation assays. **C** Representative images (left) and quantitative analysis (right) of wound healing (scratch) assays at 0 and 12 hours

(scale bar 200 μm). **D** Representative images (left) and quantitative analysis (right) of transwell migration assays (scale bar 100 μm). **E** Representative Western blot images (left) and corresponding densitometric quantification of SCARB1 protein expression (right) in orthotopic tongue tumor tissues. **F** Representative Hematoxylin and Eosin (H&E) stained sections of the orthotopic tongue tumors from the respective groups(scale bar 1mm, 100 μm). Data are presented as mean ± SD from at least three independent experiments. Statistical significance was determined by unpaired Student's t-test (A and E) or one-way ANOVA (B, C and D) followed by appropriate post-hoc tests. ns, not significant; $**p < 0.01$, $***p < 0.001$.
