# Supplementary material for: Depression-induced systemic C16-ceramide deficiency promotes OSCC via attenuation of mitochondrial apoptosis
Source: Cell Mol Life Sci. 2026 Jun 6;83(1):303. doi: 10.1007/s00018-026-06267-1 (PMC13469040; doi:10.1007/s00018-026-06267-1)

Supplementary Material: Original Uncropped Western Blots

Figure 3D

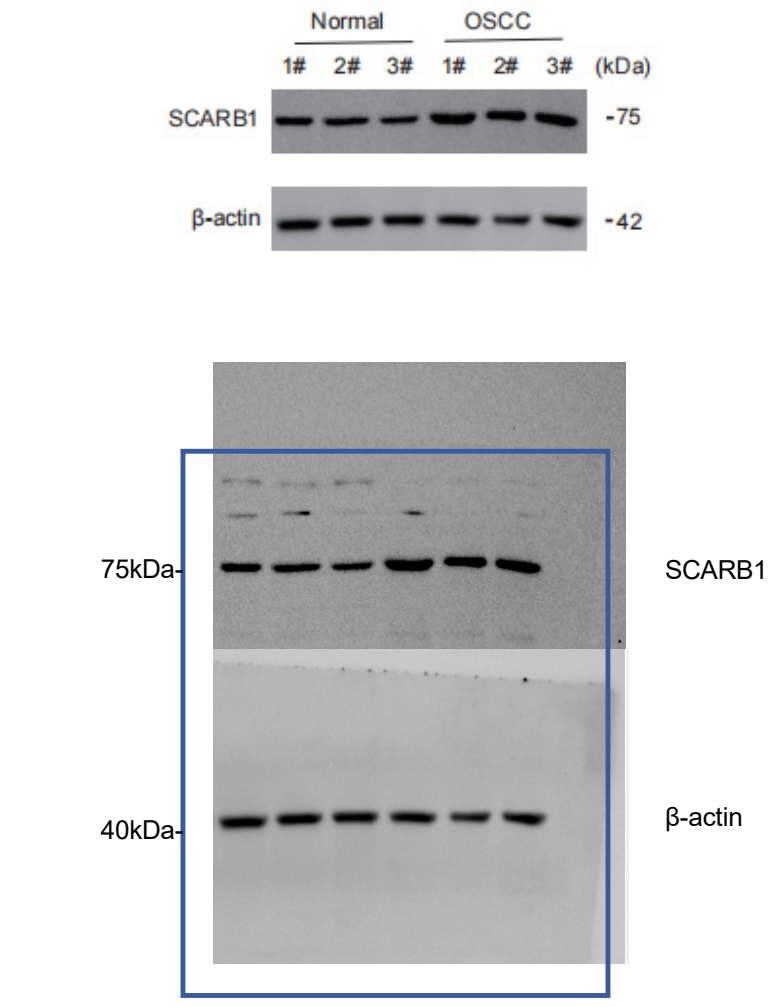

Supplementary Figure 6A

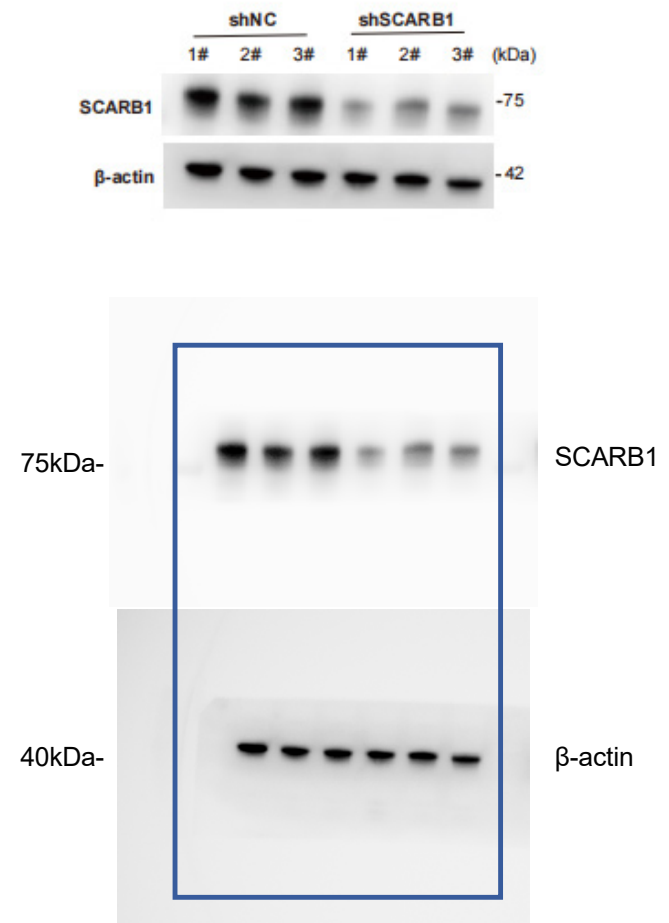

Supplementary Figure 6E

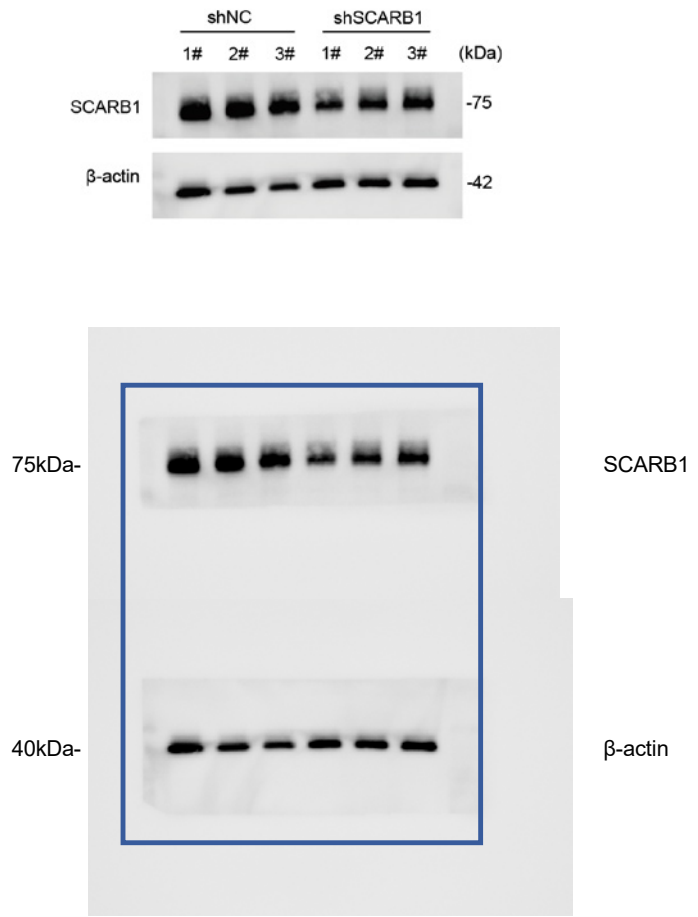

Figure 4K

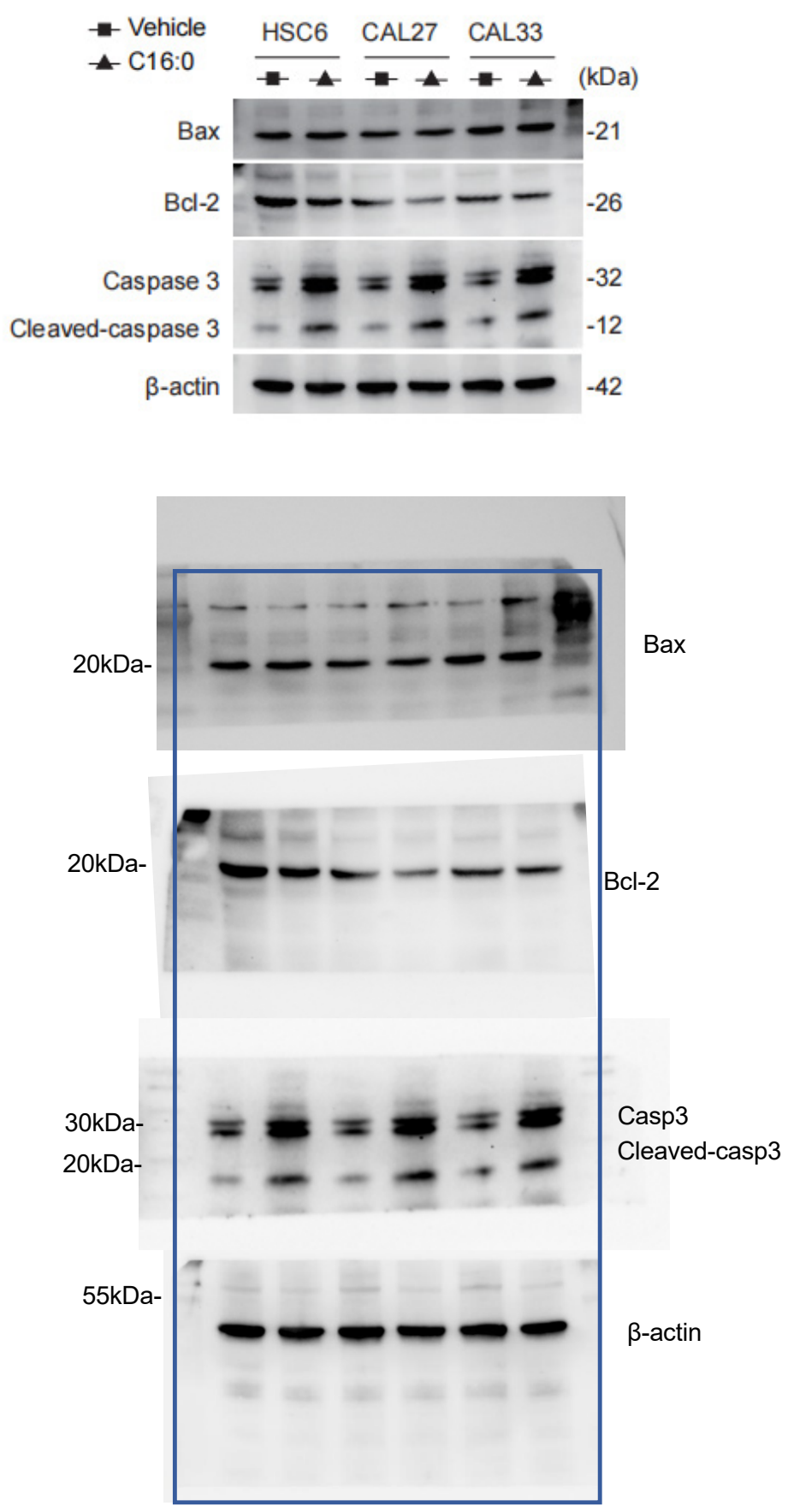

Figure 4K（two additional independent biological replicates across different cell lines）

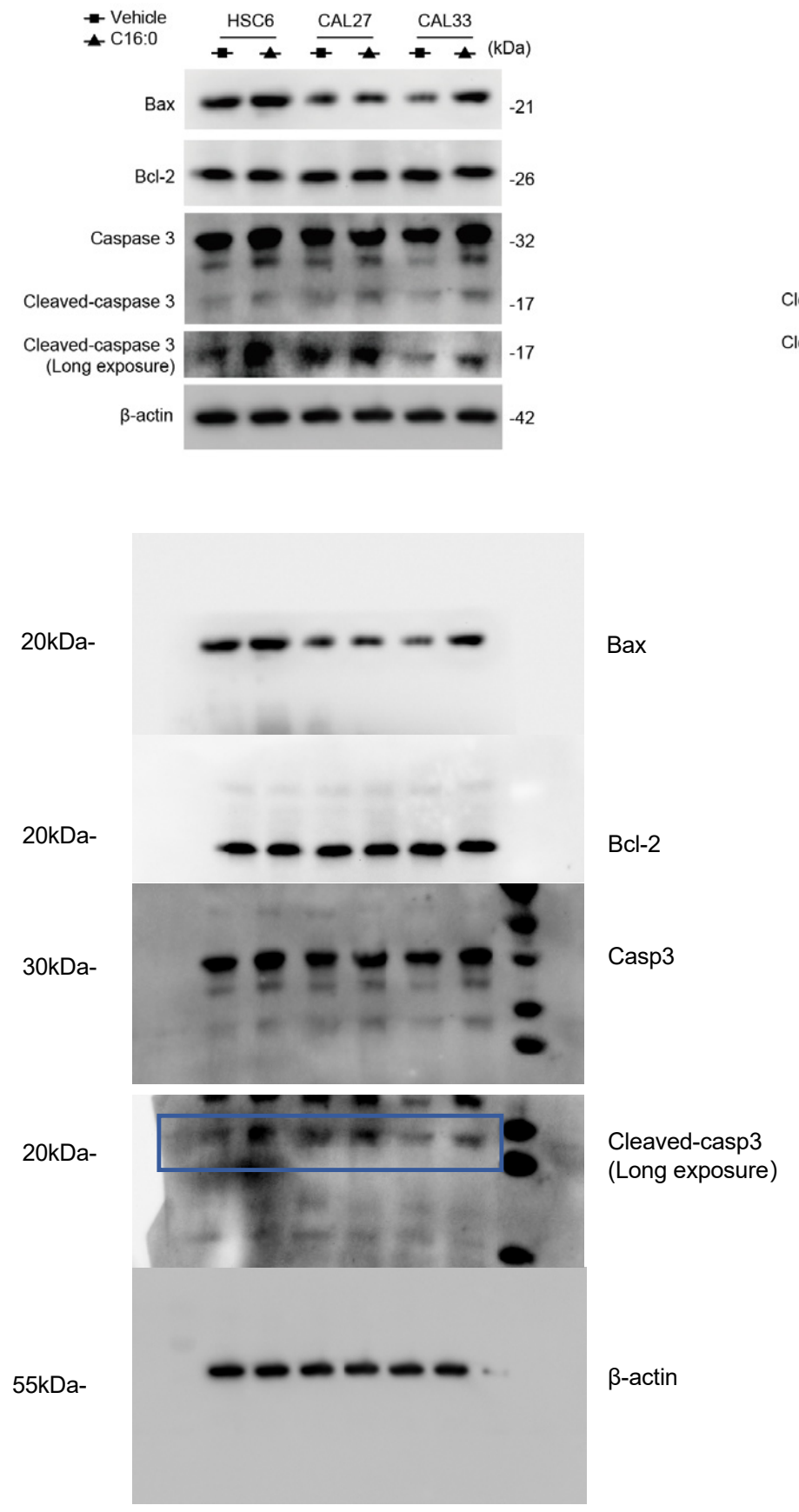

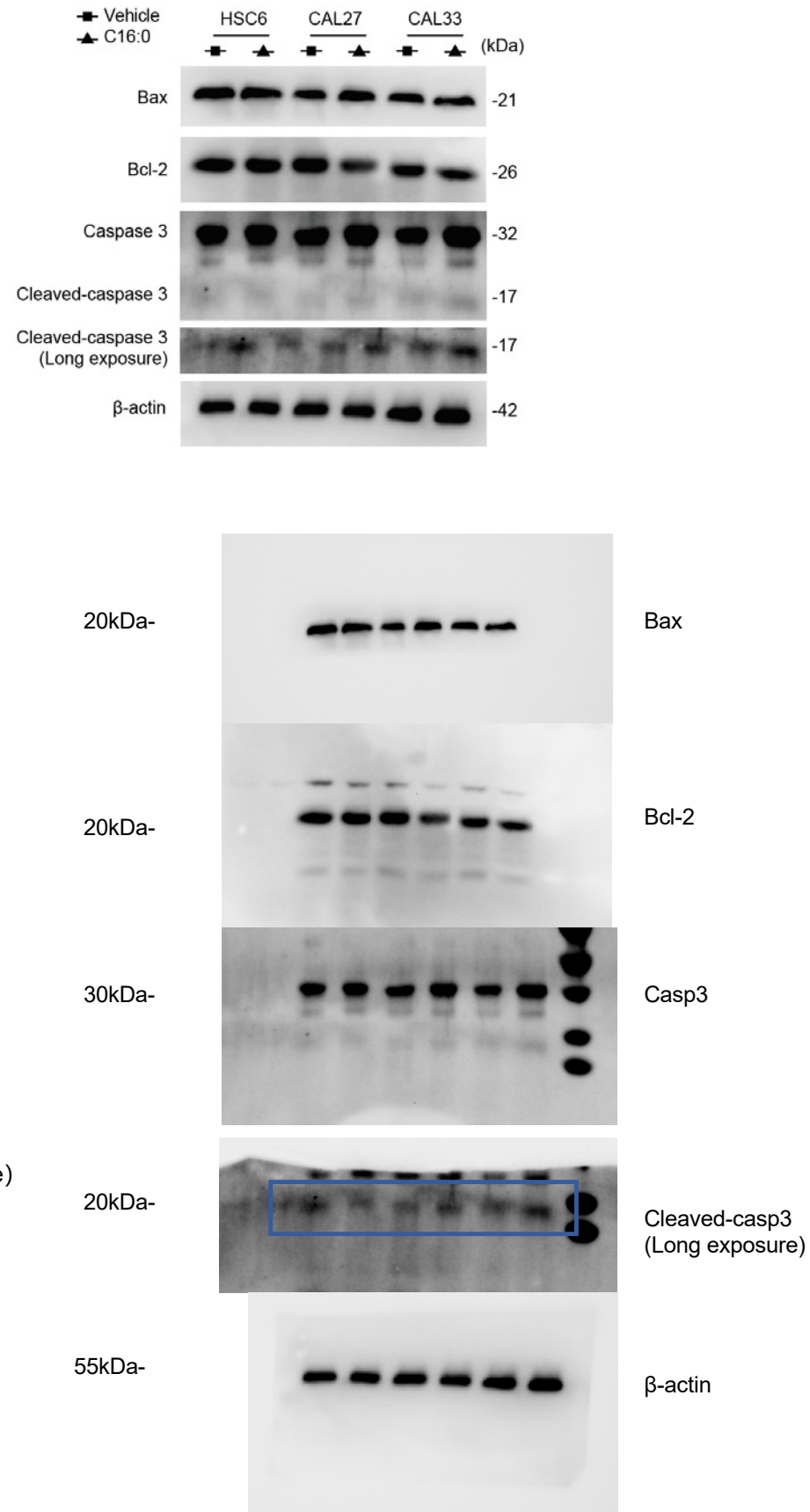

Supplement: Supplementary file 2 [file 18_2026_6267_MOESM3_ESM.pdf]
